# Multi‐omic analysis reveals genes and proteins integral to bioactivity of Echinochrome A isolated from the waste stream of the sea urchin industry in Aotearoa New Zealand

**DOI:** 10.1002/fsn3.4140

**Published:** 2024-04-02

**Authors:** Joseph Hammond, Isabella M. Das, Ruihana Paenga, Manu Caddie, Damian Skinner, Jeffrey P. Sheridan, Matthew R. Miller, Andrew B. Munkacsi

**Affiliations:** ^1^ School of Biological Sciences Victoria University of Wellington Wellington New Zealand; ^2^ Wellington High School Wellington New Zealand; ^3^ Hikurangi Bioactives Limited Partnership Ruatōria New Zealand; ^4^ Cawthron Institute Nelson New Zealand; ^5^ Centre for Biodiscovery Victoria University of Wellington Wellington New Zealand

**Keywords:** antioxidant activity, echinochrome, functional genomics, high‐throughput microscopy, iron metabolism, marine natural product, sea urchin

## Abstract

*Evechinus chloroticus* (commonly known as kina) is a sea urchin species endemic to New Zealand. Its roe is a culinary delicacy to the indigenous Māori and a globally exported food product. Echinochrome A (Ech A) is a bioactive compound isolated from the waste product of kina shells and spines; however, the molecular mechanisms of Ech A bioactivity are not well understood, partly due to Ech A never being studied using unbiased genome‐wide analysis. To explore the high‐value pharmaceutical potential of kina food waste, we obtained unbiased functional genomic and proteomic profiles of yeast cells treated with Echinochrome A. Abundance was measured for 4100 proteins every 30 min for four hours using fluorescent microscopy, resulting in the identification of 92 proteins with significant alterations in protein abundance caused by Ech A treatment that were over‐represented with specific changes in DNA replication, repair and RNA binding after 30 min, followed by specific changes in the metabolism of metal ions (specifically iron and copper) from 60–240 min. Further analysis indicated that Ech A chelated iron, and that iron supplementation negated the growth inhibition caused by Ech A. Via a growth‐based genome‐wide analysis of 4800 gene deletion strains, 20 gene deletion strains were sensitive to Ech A in an iron‐dependent manner. These genes were over‐represented in the cellular response to oxidative stress, suggesting that Ech A suppressed growth inhibition caused by oxidative stress. Unexpectedly, genes integral to cardiolipin and inositol phosphate biosynthesis were required for Ech A bioactivity. Overall, these results identify genes, proteins, and cellular processes mediating the bioactivity of Ech A. Moreover, we demonstrate unbiased genomic and proteomic methodology that will be useful for characterizing bioactive compounds in food and food waste.

## Graphical abstract

- The mechanism of action of Echinochrome A (Ech A) was evaluated using unbiased genomic and proteomic analyses.
- Growth analyses of gene deletions and measurements of protein abundance identified the importance of 20 genes and 92 proteins.
- The antioxidant activity of Ech A may begin with alterations in DNA replication, followed by iron chelation and disrupted cardiolipin metabolism.

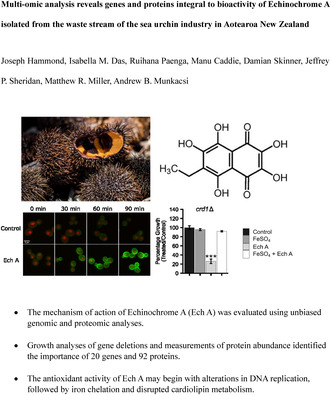

## 1 INTRODUCTION

Historically, natural products have played a key role in the treatment of human diseases and illness as active components of traditional and pharmaceutical medicines. From 1981 to 2019, 64% of approved drugs were natural products, derived from natural products or mimics of natural products (Newman & Cragg, 2020). The marine ecosystem (oceans) covers approximately 70% of the earth's surface, containing extraordinary organisms that are unique to those ecosystems. Albeit not as well studied as land plants, the marine ecosystem is a source of diverse organisms that contain unique bioactive compounds (Haefner, 2003). Indeed, the investigation of marine natural products has increased in recent years, with 1490 new marine natural products being reported in 2017 compared to only 332 in 1984 (Carroll et al., 2023). This diversity of organisms provides the opportunity to discover bioactive compounds with unique chemical features, resulting in the marine environment being a top spot for the identification of new drug leads.

**FIGURE 1:**
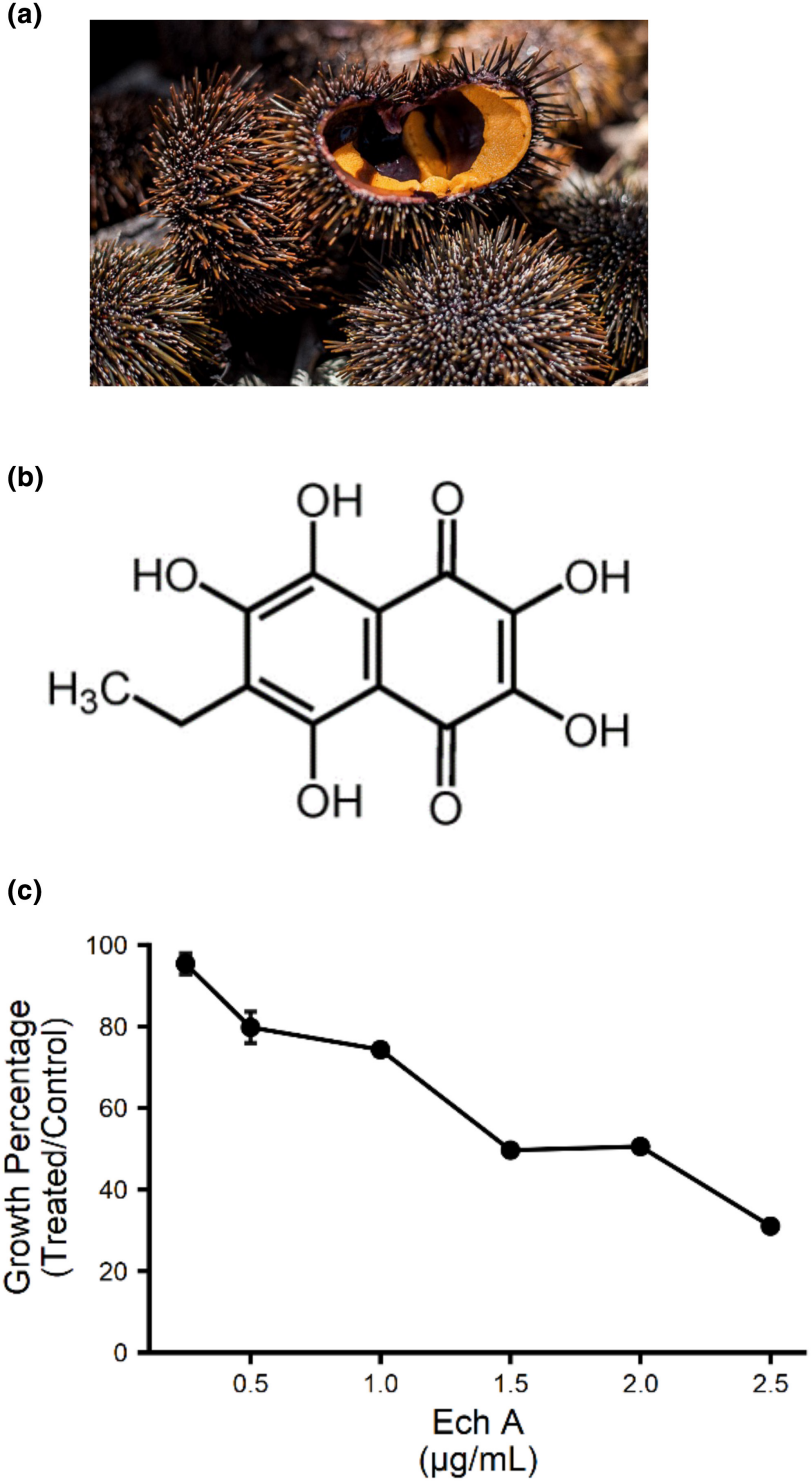
(a) Representative image of kina (*Evechinus chloroticus*) including the spines that are the source of Ech A and the roe that is a culinary delicacy and international export. (b) The chemical structure of Ech A. (c) Ech A is bioactive in yeast at a dose‐dependent manner. Growth percentage of wildtype (BY4741) in the presence of increasing concentrations of Ech A at mid‐log. Data shown as mean of technical triplicates ± *SD*.

*Evechinus chloroticus* (commonly known as kina) is a species of sea urchin endemic to New Zealand (Figure 1a). The edible roe of the kina is considered a delicacy to Māori, the indigenous people of New Zealand. This results in the commercial and recreational harvesting of ~1875 tonnes of kina per year in New Zealand (*Fisheries Infosite: Catch analysis of kina in New Zealand*, 2022). However, the shell and spines of the kina that comprises the great majority of the biomass are not edible and considered a waste product. Given immobile marine organisms (e.g., sponges) produce high levels of chemical diversity as a mode of defense, with multiple approved drugs on the market being either natural products or derivatives of sponges (Carroll et al., 2023; Newman & Cragg, 2020), it is reasonable to expect high and potent chemical diversity in kina shells. Echinochrome A (Ech A; 6‐ethyl‐2,3,5,7,8,‐pentahydroxy‐1,4‐naphthoquinone; Figure 1b) is a bioactive compound extracted from the shells and spines of sea urchins (Hou et al., 2019). Ech A has exhibited in vitro and in vivo bioactivity in multiple tissues including kidney (Cui et al., 2022), skin (Choi et al., 2022; Kim et al., 2023; Seol et al., 2021; Yun et al., 2021), heart (Artyukov et al., 2020; Jeong, Kim, Song, Lee, et al., 2014; Kim et al., 2015; Song et al., 2022; Tang et al., 2023), bone (Hou et al., 2020), lung (Lebed'ko et al., 2015; Seo et al., 2015) and eyes (Lennikov et al., 2014). These results may help explain the therapeutic efficacy of Ech A, perhaps via the antioxidant, anti‐inflammatory and metal‐chelating activities, against several common ailments and diseases (e.g., diabetes (Mohamed et al., 2016; Pham et al., 2023), ischemia/stroke (Kim et al., 2019; Sedova et al., 2015), viral infections (Fedoreyev et al., 2018; Mishchenko et al., 2020), bacterial infections (Sadek et al., 2021), inflammation (Park et al., 2021; Rubilar et al., 2021; Sadek et al., 2021), cancer (Lazarev et al., 2016; Mohamed, 2021) and neurodegenerative diseases (Ekimova et al., 2018; Lee, Pronto, et al., 2014)). As only genes involved in these bioactivities have been investigated (e.g., GSH and SOD genes to explain antioxidant bioactivity; ERK and p53 genes to explain cell survival signaling bioactivity), The genes and pathways mediating these bioactivities are not fully understood.

An unbiased genome‐wide analysis of Ech A has yet to be performed. Via the availability of two genome‐wide libraries, the genetic model Baker's yeast (*Saccharomyces cerevisiae*) has been used to determine the mechanism of action of thousands of bioactive compounds (Hillenmeyer et al., 2008; Lee, St Onge, et al., 2014; Molimau‐Samasoni et al., 2021; Parsons et al., 2006). First, growth of each deletion strain in a genome‐wide deletion library (i.e., comparing growth of the treated and untreated deletion strains) distinguishes gene deletion strains sensitive to a compound, which indicates that these genes are essential for the metabolism of the compound. This methodology was notably used to identify the antifungal mode of action of turbinmicin isolated from a sea squirt microbiome (Zhang et al., 2020). Second, the abundance of GFP‐tagged proteins in single cells can be measured in a proteome‐wide library (where each strain has a different protein C‐terminally fused to a GFP under the control of the endogenous promoter) (Huh et al., 2003). The inclusion of cell markers for nuclei and cytoplasm integrated into the genome made it possible to use high‐throughput microscopy and automated image analysis software to identify and segment individual cells for single cell analysis of protein abundance (Bircham et al., 2011). Together, the cell markers and GFP‐tagged proteins allow for researchers to monitor protein abundance changes over time in live cells, which is an ideal tool to study the changes to the proteome and provide additional insight into the mode of action of bioactive compounds caused by drug treatment (Chong et al., 2015; Kraus et al., 2017; Tkach et al., 2012).

As high‐throughput methodology exists in yeast to identify genes and proteins sensitive to thousands of compounds (Chong et al., 2015; Hillenmeyer et al., 2008; Kraus et al., 2017; Lee, St Onge, et al., 2014; Parsons et al., 2006; Tkach et al., 2012), these approaches have not been used to define the mode of action of Ech A in yeast. Here we conduct unbiased multi‐omic (genomic and proteomic) analyses in yeast to identify key genes and proteins via the gene deletion and GFP‐tagged libraries, respectively, to detect suspected as well as unsuspected pathways integral to EchA bioactivity. Our study thus demonstrates unbiased genomic and proteomic methodology that will be useful for characterizing bioactive compounds in food and food waste.

## 2 MATERIALS AND METHODS

### 2.1 Compound extraction

Ech A was extracted from 25 freeze‐dried kina (*Evechinus chloroticus*) shells collected between 30/9/2018 and 28/10/2018 by hapū (Māori subtribe)‐appointed collectors in seven sites across Tairāwhiti on the East Coast of New Zealand. The kina were collected from intertidal regions by hand and were frozen until extraction. The extraction was conducted in the dark with minimal UV exposure. The harvested kina shells were freeze‐dried and crushed into a very fine powder. In a 5 L conical flask containing 1 L of 6 M HCl (1:1 concentrated HCl and water), 400 g of kina shell powder was added into the flask in 30 g stages and filtered, followed by the addition of 800 mL of ethyl acetate and 200 mL of brine. This was then separated using a separation funnel, washed twice with water to remove any acid, washed once with sodium sulphate, transferred into a large round bottom flask, and dried the rotary in vacuo at 20°C. The powder was then suspended in dichloromethane, washed with water, dried in 20°C rotary in vacuo, resuspended in absolute ethanol, and stored in aluminum foil‐wrapped tubes at −20°C. A nitrogen blanket was applied after each use and before storage at −20°C to minimize any degradation of the compound due to oxidation and minimize any evaporation of the ethanol.

### 2.2 Strains

The *Saccharomyces cerevisiae* strains were derived from the S288C genetic background. Single gene deletion strains in the deletion library (Open Biosystems) were in the background of BY4741 (MATa ura3Δ0 leu2Δ0 his3Δ1 met15Δ0) where each gene was replaced with a kanamycin‐resistance gene. GFP/RFP‐tagged strains (MATa xxx‐GFP::HIS5 can1Δ::STE2pr‐Sp_LEU2; lyp1Δ::HPH::NLS‐RS2::TEF2pr_mCherry; his3Δ1 leu2Δ0 ura3Δ0 met15Δ0 LYS2) were previously constructed (Bircham et al., 2011).

### 2.3 Media

Synthetic Complete (SC) and yeast extract peptone dextrose (YPD) media were prepared as previously described (Molimau‐Samasoni et al., 2021). All media reagents were purchased from Formedium.

### 2.4 Liquid‐based bioactivity

Bioactivity was assessed by quantifying the growth of yeast as previously described (Molimau‐Samasoni et al., 2021). Single colonies were incubated overnight in 2 mL of SC broth at 30°C with rotation, and subcultured to generate 1 mL of each strain at 0.128 × 10^7^ cells/mL. The desired Ech A concentrations were prepared in SC broth with a final concentration of ethanol being 1% across all concentrations. In a 96‐well plate (Jet Biofil), 95 μL of the Ech A + SC stocks were added to the wells in triplicate, to which 5 μL of the subcultured cells were added to the wells to give a final volume of 100 μL in each well. The control media contained only SC broth with 1% ethanol. Plates were incubated in the dark at 30°C, and absorbance (OD_590_) readings were recorded using an Envision plate reader (Perkin‐Elmer) at the start of the experiment (t0) as well as at mid‐log growth for the untreated cells. The absorbance measurements were normalized by subtracting the initial t0 absorbance. Growth percentage (the ratio of growth in treated and untreated cells) was calculated using the following formula: Growth Percentage (%) = (Absorbance (Treated)/Absorbance (Control)) × 100.

### 2.5 High‐throughput confocal time‐lapse microscopy

The abundance of GFP‐tagged proteins was measured using high‐throughput microscopy as previously described (Tkach et al., 2012) with the addition of a time‐lapse component. For each strain in the GFP library, cells were pinned from agar in a 384‐colony format into 96‐well plates (Jet Biofil) containing 200 μL SC broth, grown overnight at 30°C, shaken and diluted to approximately 5 × 10^5^ cells/mL. Then 30 μL of each strain was aliquoted into two wells of a 384‐well Cell Carrier Ultra optical clear plate (Perkin‐Elmer), accounting for control and treated wells on the same plate. The optical plate was incubated for 2 h at 30°C, followed by the addition of 20 μL of SC + 2.5% ethanol/tyloxapol (Sigma) (4:1) with and without Ech A to achieve a final concentration of 1% ethanol/tyloxapol and a final Ech A concentration of 2 μg/mL that elicited 50% growth inhibition in control cells. The optical plate was then shaken, left to settle for 10 min, incubated at 30°C in the InCell Analyzer 6500 HS (General Electric) for 4 h, and imaged every 30 min at 100% laser power. The GFP channel had an exposure time of 800 ms, with the excitation at 488 nm and an emission filter of 524 nm. The RFP channel had an exposure time of 2000 ms, with the excitation at 561 nm and the emission filter of 605 nm.

### 2.6 Image analysis

Images captured using high‐throughput microscopy were analyzed for fluorescent abundance of GFP relative to RFP as previously described (Bircham et al., 2011). The captured RFP images were corrected using a Gaussian blur and exposure contrast correction, and then converted to 8‐bit images using Fiji (Schindelin et al., 2012) to decrease the signal‐to‐noise ratio between the cells and the background in order to increase the accuracy of cell segmentation. The corrected images and the original images were processed through a custom CellProfiler pipeline (McQuin et al., 2018) for segmentation and analysis. Fluorescent cells were segmented using a bright nuclear red fluorescent protein Redstar2 and the dimmer cytoplasmic mCherry. Only cells with a single nucleus and cytoplasm were masked and cropped for analysis. The GFP fluorescence intensity was measured for each cell and normalized to the RFP intensity. Z‐scores were calculated using relative fluorescence for each strain, treatment and time point by the population average to determine changes in protein abundance. Strains that scored a z‐score greater than 10 were classified with an increase in protein abundance and z‐scores less than −10 were classified with a decrease in protein abundance.

### 2.7 Iron rescue assay

Iron supplementation was used to evaluate intracellular iron levels as previously described (Shakoury‐Elizeh et al., 2010). Cells were incubated overnight at 30°C in 2 mL of SC broth, and then diluted to a 1 mL subculture with a concentration of approximately 0.128 × 10^7^ cells/mL. A range of Ech A concentrations were prepared in 500 μL of SC broth in the presence or absence of FeCl_3_ (Sigma) or FeSO_4_ (Sigma) as well as iron‐free SC broth with a final concentration of 1% ethanol across all treatments. In a 96‐well plate (Jet Biofil), 95 μL of each treatment were added to the wells in triplicate, followed by the addition of 5 μL of the subculture to give a final volume of 100 μL in each well. The plate was incubated in the dark at 30°C. Absorbance readings were recorded at a wavelength of 590 nm using an Envision plate reader (Perkin‐Elmer) at the start of the experiment as well as the time point where the untreated cells were at mid‐log. Absorbance was normalized by subtracting the initial t0 and growth percentage was calculated for each treatment compared to the control in the same media with or without iron.

### 2.8 Iron chelation analysis

The cell‐free colorimetric chrome azurol S (CAS) assay was used to measure iron chelation (Alexander & Zuberer, 1991). The CAS assay solution was prepared by mixing 750 μL of 1 mM FeCl_3_ in 10 mM HCl with 3.75 mL of 2 mM CAS in a 50 mL volumetric flask, followed by the addition of 25 mL of 0.92 M MES monohydrate (2‐(N‐morpholino) ethane sulfonic acid) buffer (pH 5.6) (Sigma) that was added dropwise to the volumetric flask. Then 10.95 mg of HDTMA (hexa‐decyl‐trimethyl‐ammonium bromide) (Sigma) dissolved in 12.5 mL of ddH_2_0 was added and further diluted to 50 mL with ddH_2_O. Next 95 μL of the prepared CAS assay solution was aliquoted to each well in a 96‐well plate (Jet Biofil), followed by the addition of 5 μL of varying concentrations of Ech A or the iron chelator ethylenediaminetetraacetic acid (EDTA) (Thermo Fisher). Images and absorbance readings (630 nm) were taken at the start of the experiment and after 2 h of incubation in the dark at room temperature.

### 2.9 Agar‐based bioactivity assay

Bioactivity in agar was determined by measuring growth in treated cells compared to untreated cells (Parsons et al., 2006). Using a 24‐well plate (Jet Biofil), 1 mL of molten SC agar was added to each well containing Ech A for the desired concentrations and slowly resuspended with a pipette. The control and treatments all contained 1% ethanol. The plates were then left to set for 1 h in the dark and then dried for 15 min in a laminar flow hood (Holten HB2460 LaminAir). An overnight culture of BY4741 was diluted to three different concentrations of cells (1 × 10^8^, 1 × 10^6^ and 1 × 10^4^ cells/mL). Then 2 μL of each yeast concentration was spotted on agar in each well, incubated at 30°C for 24 h in the dark, and imaged using a digital camera (Canon EOS 600D).

### 2.10 Chemical genomic analysis

Growth of each strain in the deletion library was measured as previously described (Parsons et al., 2006), here in the presence and absence of EchA. A single plate of the yeast deletion library was selected at random to identify a concentration that conferred 10%–20% growth inhibition in 1536‐colony format (384 colonies in quadruplicate). The ROTOR HDA (Singer Instruments) was used to replicate the plate on SC agar with either a specific concentration of Ech A or the 1% ethanol vehicle control. Plates were incubated at 30°C for 24 h in the dark, imaged with a digital camera (Canon EOS 600D), and processed using SGA Tools (Wagih et al., 2013) where colony size was normalized for growth across the plate and growth inhibition was calculated for each strain via the ratio of growth on treated and control plates. The entire deletion library was then grown in the presence and absence of the optimized concentration. Plates were incubated at 30°C for 24 h in the dark, imaged with a digital camera (Canon EOS 600D), and processed using SGA Tools (Wagih et al., 2013) where colony size was normalized for growth across the plate and growth inhibition was calculated for each strain via the ratio of growth on treated and control plates. The treated colonies in quadruplicate were compared to the quadruplicate control colonies in order to calculate the z‐scores representing the growth inhibition for each strain. Deletion strains that were resistant (negative z‐score) or sensitive (positive z‐score) with a *p*‐value < .05 were selected for validation. Validation was conducted via growth comparisons of serial dilutions of each strain that were grown in the same conditions as the genome‐wide analysis and imaged using a digital camera (Canon EOS 600D) after 24 h of incubation at 30°C in the dark.

### 2.11 Antioxidant activity

Growth in the presence and absence of hydrogen peroxide (H_2_O_2_) was used to measure antioxidant activity as previously described (James et al., 2015). Cells were incubated overnight at 30°C in 2 mL of SC broth, diluted to a 1 mL subculture with a concentration of approximately 0.128 × 10^7^ cells/mL, and then grown with and without 1.5 mM H_2_O_2_ and 1 μg/mL Ech A where all treatments contained 1% ethanol. In a 96‐well plate (Jet Biofil), 95 μL of each treatment was added to the wells in triplicate, followed by the addition of 5 μL of the subculture to give a final volume of 100 μL in each well. The plate was incubated in the dark at 30°C. Absorbance readings were recorded at a wavelength of 590 nm using an Envision plate reader (Perkin‐Elmer) at the start of the experiment (t0) as well as the time point where the untreated cells were at mid‐log. Absorbance was normalized by subtracting the initial t0 and the residual growth was calculated for each treatment compared to the control.

## 3 RESULTS

### 3.1 Ech A is bioactive in yeast in a dose‐dependent manner

Growth inhibition of yeast cells is regularly used to determine the bioactivity of compounds and extracts (Hillenmeyer et al., 2008; Lee, St Onge, et al., 2014; Molimau‐Samasoni et al., 2021; Parsons et al., 2006). To assess the bioactivity of Ech A in the yeast model organism, the residual growth of wildtype (BY4741) was measured in a liquid‐based growth assay. Growth was measured at t0 (time point 0 h) and a time point during the exponential growth phase where untreated cells of each strain had reached mid‐log. Between Ech A concentrations ranging from 0.5–5 μg/mL, both BY4741 exhibited a reduction in growth, in a dose‐dependent manner with 20%–70% growth inhibition (Figure 1c). These results determined that Ech A is bioactive in yeast cells, which provides the foundation to take advantage of the genomic and proteomic tools available in yeast.

### 3.2 Ech A alters abundance of 92 proteins in a low‐throughput analysis

**FIGURE 2:**
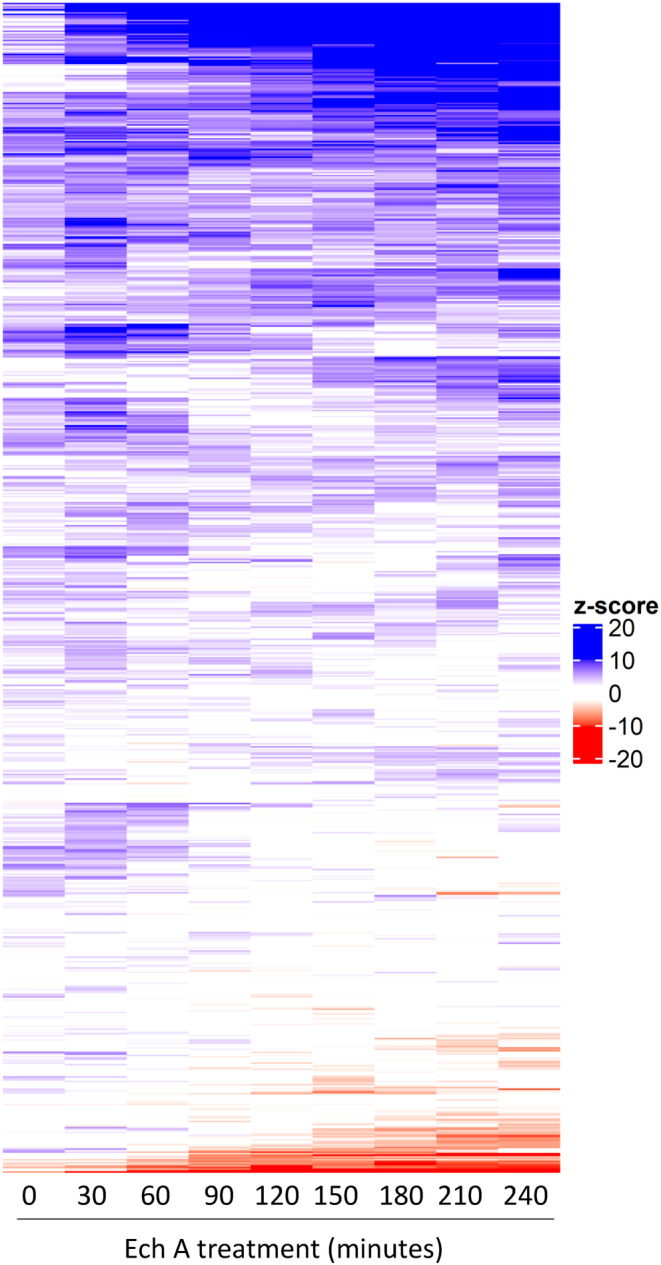
Ech A alters abundance of 92 proteins during the 4‐h time‐lapse. Heatmap represents the z‐score (ratio of fluorescence in treated and untreated cells) of each protein at every timepoint (30 min) over 4 h. Increased and decreased abundance is illustrated with shades of blue and red, respectively.

Cells detect and respond to environmental changes by regulating protein abundance (Bircham et al., 2011; Kraus et al., 2017; Tkach et al., 2012). Using a library of ~4100 strains that have a green fluorescent protein (GFP) fused to a different open reading frame, we investigated protein abundance in response to Ech A treatment at a genome‐wide level. The entire library was treated with and without 2 μg/mL Ech A, incubated at 30°C, and images were captured every 30 min over 4 h via high‐throughput confocal microscopy. GFP intensities were analyzed for the segmented cells and assigned z‐scores for each strain compared to the control at every time point. Specifically, 2.1% (*n* = 86) and 0.15% (*n* = 6) of the altered strains exhibited an increase and decrease in protein abundance, respectively, at any time point over the four hours (Figure 2; Table S1). Interestingly, 15 of these proteins are either localized to the mitochondria, function in the mitochondria or cause defective mitochondria when mutated (Table S2), indicating that Ech A bioactivity in yeast involves mitochondria, which would align with previous literature in mammalian cells (Jeong, Kim, Song, Lee, et al., 2014; Jeong, Kim, Song, Noh, et al., 2014). These proteins are functionally involved in the metabolism of metal ions, multidrug resistance, autophagy as well as unknown functions (representative images of significant changes in 17 proteins over the 4‐h time‐lapse are shown in Figures S1 and S2).

#### 3.2.1 Drug response

**FIGURE 3:**
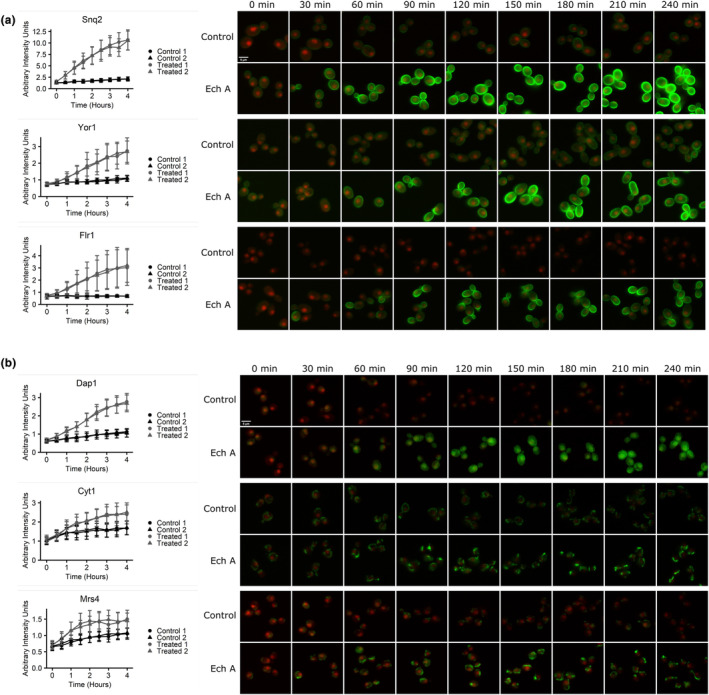
Ech A treatment significantly changes abundance of proteins involved in (a) drug response and (b) the metabolism of metal ions. GFP strains were treated with and without 2 μg/mL Ech A and imaged every 30 min over 4 h. Each strain was normalized to the RFP in the control. Line graphs of each strain with the mean arbitrary intensity units (representing GFP fluorescence) of each control and treated replicate over the 4‐h time‐lapse ± *SD*.

Of the 92 proteins with altered abundance, there were notable changes in proteins involved in multidrug resistance with statistically significant changes in abundance in five proteins (Table S3). SNQ2 is an ATP‐binding cassette multidrug transporter localized to the plasma membrane of the cell and involved in multidrug resistance mechanisms; this protein showed the greatest increase in protein abundance over the 4‐h time‐lapse (z‐score = 51.25) (Figure 3). Other genes involved in multidrug resistance that also showed an increase in protein abundance were YOR1, FLR1, YLR179C, and CIS1 (Figure 3; Table S3). YOR1 is a plasma membrane ATP‐binding multidrug transporter which had an increase in protein abundance from 90–240 min. FLR1 is another plasma membrane multidrug transporter that had an increase in protein abundance from 90–240 min. YLR179C and CIS1 both have unknown functions however both open reading frames are activated by transcription factors that are part of the multidrug resistance system in a yeast cell. These results reflect the response of the yeast cells to Ech A via specific changes in the abundance of proteins involved in the multidrug resistance mechanism.

#### 3.2.2 Metal ion metabolism

Eight strains that exhibited statistically significant changes in abundance are all involved in metal ion metabolism (Figure 3; Table S4). DAP1 is a heme‐binding protein involved in the regulation of cytochrome protein Erg11 and acts as a damage response protein; this protein was significantly increased in protein abundance at every time point from 30 min onwards. CYT1 expression is regulated by the heme‐activated protein Hap2, and is a component of the mitochondrial respiratory chain; this protein increased in protein abundance from 150–240 min. MRS4 is an iron ion transporter that transports Fe^2+^ across the inner mitochondrial membrane and is highly active during iron deficiency; this protein was significantly increased in protein abundance at every time point from 60 min onwards. ARN1 is a transporter protein that is responsible for uptake of bound iron; this protein was increased at every time point from 60 min onwards. TIS11 is a mRNA‐binding protein expressed during iron starvation that was significantly increased at every time point. SMF3 is a metal ion transporter that plays a role in iron homeostasis; it had a significant increase in protein abundance at every time point from 30 min onward. These six proteins are integral to the metabolism of iron ions. Their increase in protein abundance suggests that there is a lower concentration of metal ions available to the cell, within the cells or in the surrounding environment, due to the treatment of Ech A. Proteins involved in the metabolism of other metal ions were also observed to be altered in their abundance. CCC2 is a transporter of copper ions that had an increase in protein abundance at every time point from 60 min onwards. COT1 is a mediator of zinc transport into the vacuole that had an increase in protein abundance from 150–240 min. Having an increase in protein abundance in these eight proteins suggests that Ech A is altering the concentration of available metal ions to the cell and the increase in protein abundance is the cell trying to find and use the metal ions that are still available.

#### 3.2.3 Autophagy

A protein critically involved in autophagy also showed a significant decrease in protein abundance. SAM3 is a high‐affinity S‐adenosylmethionine permease required for the cell to use S‐adenosylmethionine as a sulfur source; when S‐adenosylmethionine is deficient, autophagy is activated (Ogawa et al., 2016). SAM3 abundance decreased with Ech A treatment as time increased, with the lowest overall z‐score of −25.78 resulting at 4 h (Table S1). This result suggests Ech A bioactivity occurs via a mechanism that includes autophagy.

#### 3.2.4 Unknown functions

Some proteins that showed among the greatest abundance changes have unknown functions (Figure S2). These include YDR476C, APD1, and YCR087C‐A. YCR087C‐A with decreases in abundance at every time point except for t0 (0 min), and the other two proteins with increases at every time point from 60–240 min. Though the function of Apd1 is not fully understood, it has been reported to be an iron‐containing thioredoxin‐like ferrodoxin involved in antioxidant activity (Stegmaier et al., 2019). The increase in protein abundance could reflect an increased concentration of hydrogen peroxide, caused by Ech A, and that the cell is trying to get rid of additional hydrogen peroxide. Alternatively, it could indicate there was a depletion of hydrogen peroxide, and that the cell has increased the protein abundance to use the available hydrogen peroxide. Though the functions of these genes are not fully understood, these changes in protein abundance show that these proteins and their associated processes in oxidative stress are involved in the metabolism of Ech A.

### 3.3 Statistical over‐representation (enrichment) of altered protein abundance in metal ion metabolism with Ech A treatment

**FIGURE 4:**
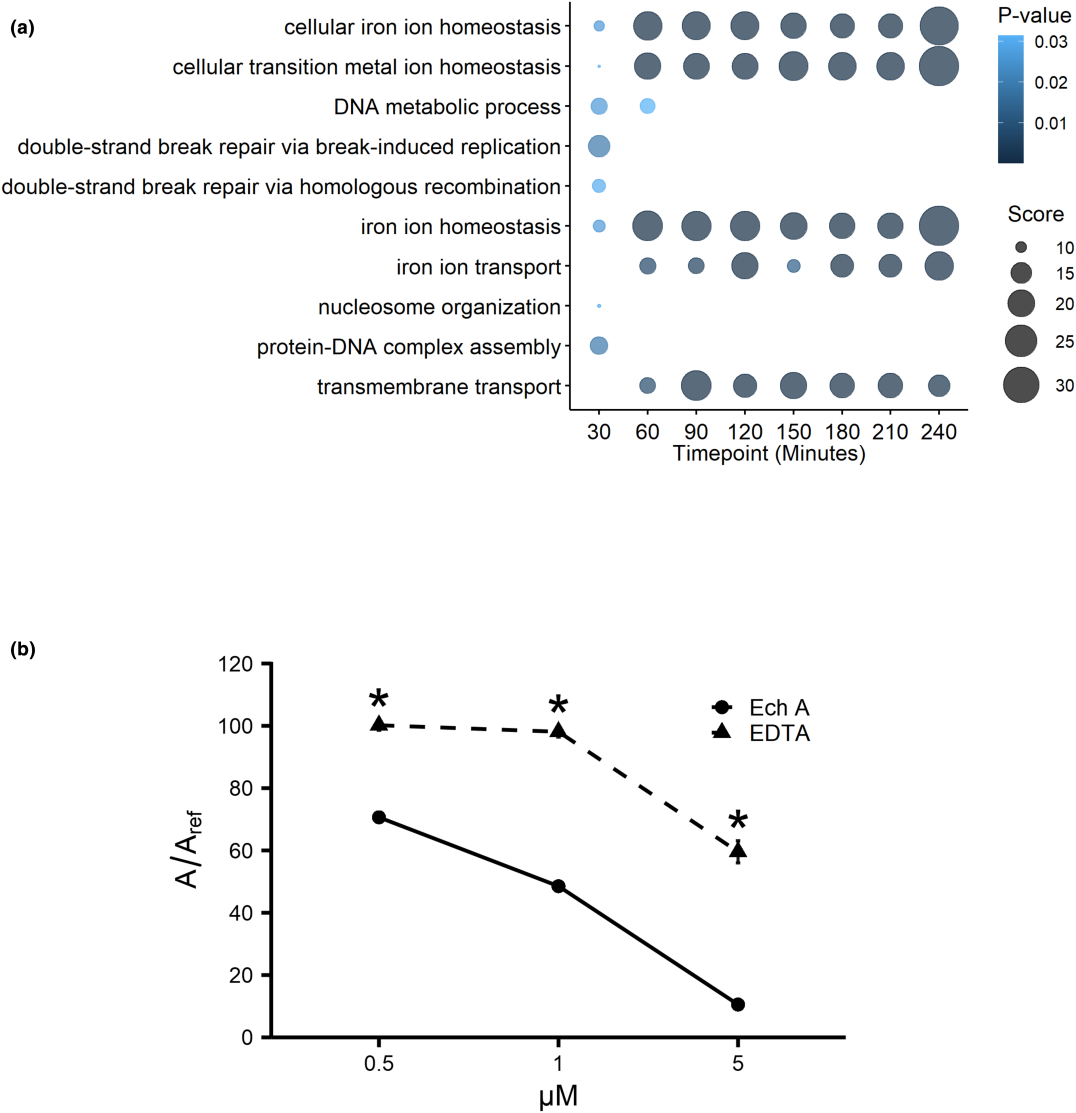
(a) Enrichment analysis of proteins altered by Ech A reveals changes in DNA repair precede changes in iron metabolism. Over‐representation of biological processes of strains with significant changes in protein abundance at each 30‐min timepoint using YeastEnrichr. Statistical enrichment is shown with *p*‐value for color and combined score for size of bubbles. (b) Iron chelation was measured using the cell‐free CAS assay at various concentrations of Ech A or EDTA (0.5, 1, and 5 μM) relative to an untreated reference (A_ref_). Absorbance (630 nm) was measured at 2 h and shown here as the mean percentage of A (Ech A or EDTA) relative to A_ref_ (the untreated reference control) ± *SD*; *p ≤ .05, student's *t*‐test comparing Ech A and EDTA at each concentration.

Enrichment analyses provide insight into the over‐representation of gene functions within a given gene set (Kuleshov et al., 2016). We used YeastEnrichr for the analysis of Gene Ontology (GO) terms to gain understanding of the biological processes and molecular functions over‐represented among the proteins with altered expression levels at each time point between 30–240 min. The 92 Ech A‐responsive proteins were enriched within the first 30 min of treatment for processes within DNA replication and repair, followed by enrichments for processes within iron ion transport and homeostasis as well as transition metal ion transport at every time point after 60 min (Figure 4a). These results corroborate the changes in iron transport proteins that were easily detected by visual inspection (Figure 3), and further emphasize the mechanism of altered iron metabolism with Ech A treatment.

### 3.4 Ech A chelates iron in a cell‐free assay

The GFP strains that increased in abundance with Ech A treatment were enriched in iron transport, suggesting that Ech A disrupts cellular iron homeostasis. To determine if Ech A chelates iron, a chrome azurol S (CAS) assay was conducted (Alexander & Zuberer, 1991). The CAS assay is a colorimetric assay that uses a solution that, when exposed to an iron chelator, changes color from blue to yellow. To quantify these results, percentages of each treatment were calculated using the absorbance (630 nm) of each treatment sample divided by the absorbance (630 nm) of the untreated reference solution (Figure 4b). The percent absorbance was not altered at 0.5 μM and 1 μM EDTA with 98%–100% ratios, and then reduced to 60% with 5 μM EDTA. In contrast, the 0.5 μM Ech A treatment resulted in 71% ratio, further reduced to 49% with 1 μM Ech A, and even further reduced to 11% with 5 μM Ech A. These results suggest Ech A chelates iron as effectively as EDTA.

### 3.5 Genome‐wide analysis identifies 36 genes involved in Ech A bioactivity

**FIGURE 5:**
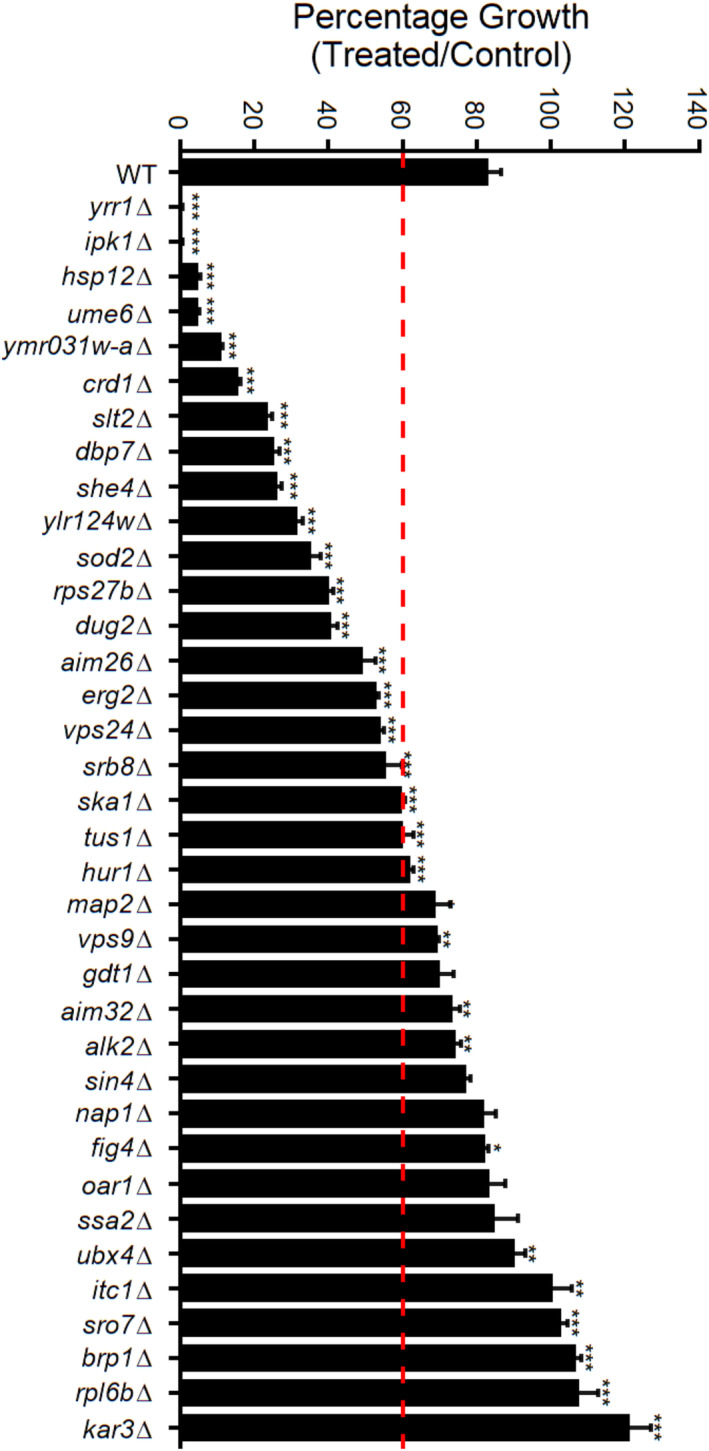
Thirty‐six genes are required for normal metabolism of Ech A. Cells were grown with and without 2 μg/mL of Ech A until control cells were at mid‐log. Percentage growth was calculated via the ratio of absorbance in treated and untreated cells at mid‐log. Red dashed line indicates 40% growth inhibition exhibited by Ech A treatment. Data shown as mean of technical triplicates ± *SD*; *p ≤ .05, **p ≤ .01, ***p ≤ .005, student's *t*‐test compared to wildtype.

The requirement of specific genes for bioactivity can be determined via the screening of gene deletion libraries. Gene deletions with impaired growth (hypersensitive) to compounds reveal the genes required to buffer the mechanism of action of the compounds (Hillenmeyer et al., 2008; Lee, St Onge, et al., 2014; Molimau‐Samasoni et al., 2021; Parsons et al., 2006). The desired concentration to use for a genome‐wide analysis would have 10%–20% growth inhibition compared to the control, which would yield an 80%–90% window to detect additional growth inhibition due to the gene deletion. Using an Ech A treatment that conferred an average 17% growth inhibition in a random subset of 384 deletion strains, we quantified growth of ~4800 strains in the presence and absence of 7.5 μg/mL Ech A in an agar‐based assay. This resulted in 166 deletion strains that had significantly altered growth (*p* < .05) with Ech A treatment. To further test the sensitivity of these 166 strains, a liquid‐based growth assay was conducted to provide robust evidence that these gene deletions strains were sensitive to Ech A in agar as well as liquid assays. Thirty gene deletion strains had significantly altered growth compared to wildtype (Figure 5; Table S5). Notably, 20 gene deletion strains exhibited more than 40% growth inhibition with the Ech A treatment. There was virtually no growth (99% growth inhibition) with deletion of the multidrug‐resistant transcription factor YRR1 or deletion of the inositol kinase IPK1. Significant (74%–95% growth inhibition) was also observed with deletion of the heat shock protein HSP12, the histone deacetylase UME6, the unknown protein YMR031W‐A, the cardiolipin synthase CRD1, the serine/threonine MAP kinase SLT2, the RNA helicase DBP7, or the actin cytoskeletal protein SHE4. These results indicate that these 20 genes and their associated functions, processes, and pathways are required for the normal metabolism of Ech A in yeast.

### 3.6 Iron supplementation rescues Ech A‐mediated growth defects

**FIGURE 6:**
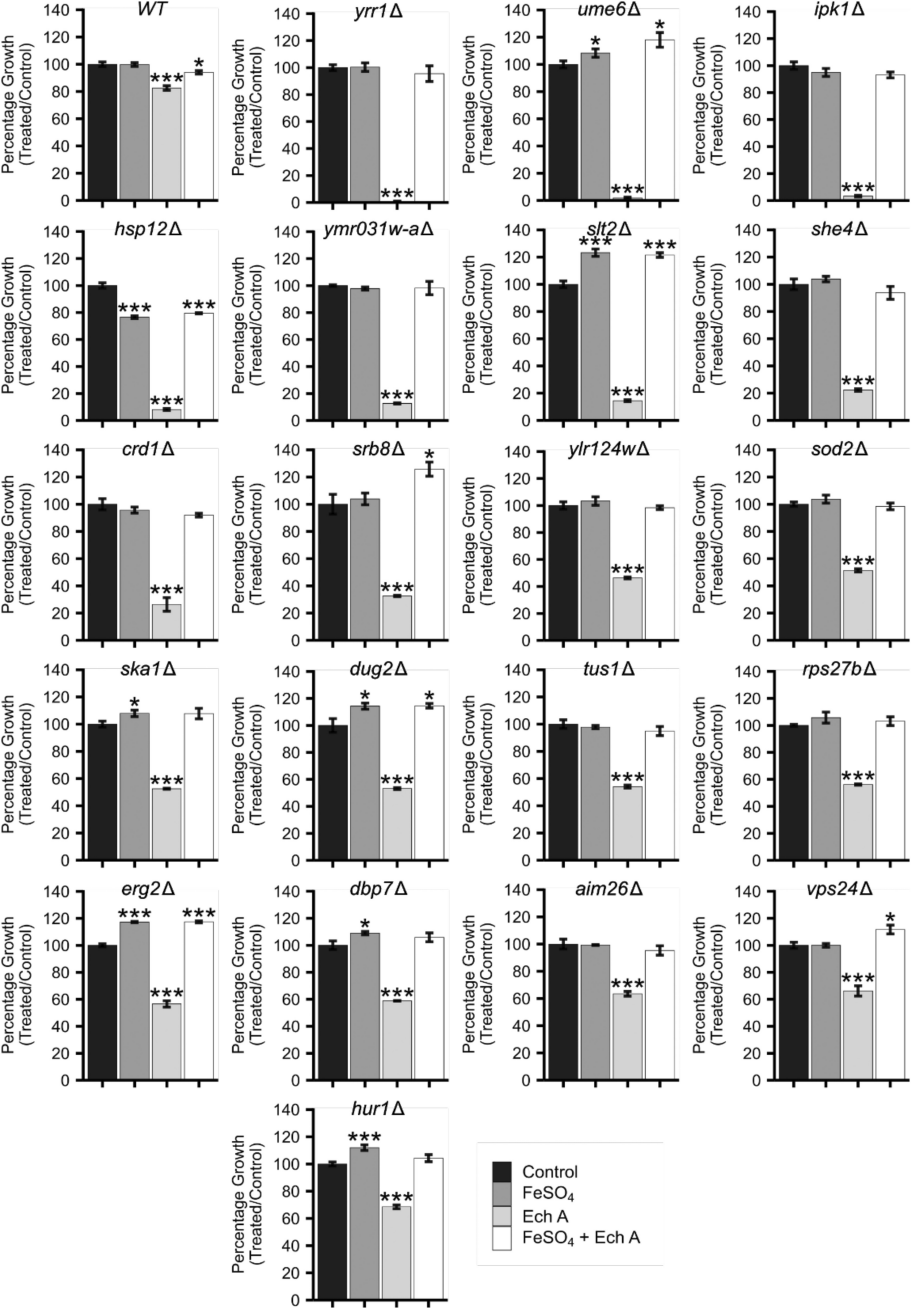
Supplementation of exogenous iron rescues growth defects caused by Ech A treatment. Cells were grown with and without 1 μg/mL of Ech A and with and without 100 μM of FeSO_4_. Percentage growth was calculated via the ratio of absorbance in treated and untreated cells at mid‐log. Data shown as mean of technical triplicates ± *SD*. *p ≤ .05, **p ≤ .01, ***p ≤ .005, student's *t*‐test comparing treated to the untreated control.

Since Ech A chelated iron (Figure 4b), we hypothesized that Ech A affects the transport of iron either into the cell or within the cell. Therefore, we expected supplementation of exogenous iron would alleviate the growth defect in cells treated with Ech A. To test this hypothesis, growth of WT and the 20 hypersensitive gene deletion strains was measured in four conditions (control media without any treatment, control media supplemented with FeSO_4_, control media supplemented with Ech A, or control media supplemented with FeSO_4_ and Ech A). The iron concentrations were chosen for optimal yeast growth as previously determined (Shakoury‐Elizeh et al., 2010). In comparison to the Ech A alone treatment that conferred a significant growth defect in WT and the 20 gene deletion strains, the co‐treatment of FeSO4 and Ech A did not exhibit a significant growth defect in WT or any gene deletion strain (Figure 6). Notably, iron supplementation completely rescued the growth of all strains regardless of how sensitive the strain was to Ech A. For example, *yrr1Δ* was practically inviable when treated with Ech A, and this strain had growth comparable to the control when co‐treated with Ech A and iron. These results indicate that iron depletion may be a major mechanism of action for Ech A bioactivity.

### 3.7 Ech A exhibits antioxidant activity

**FIGURE 7:**
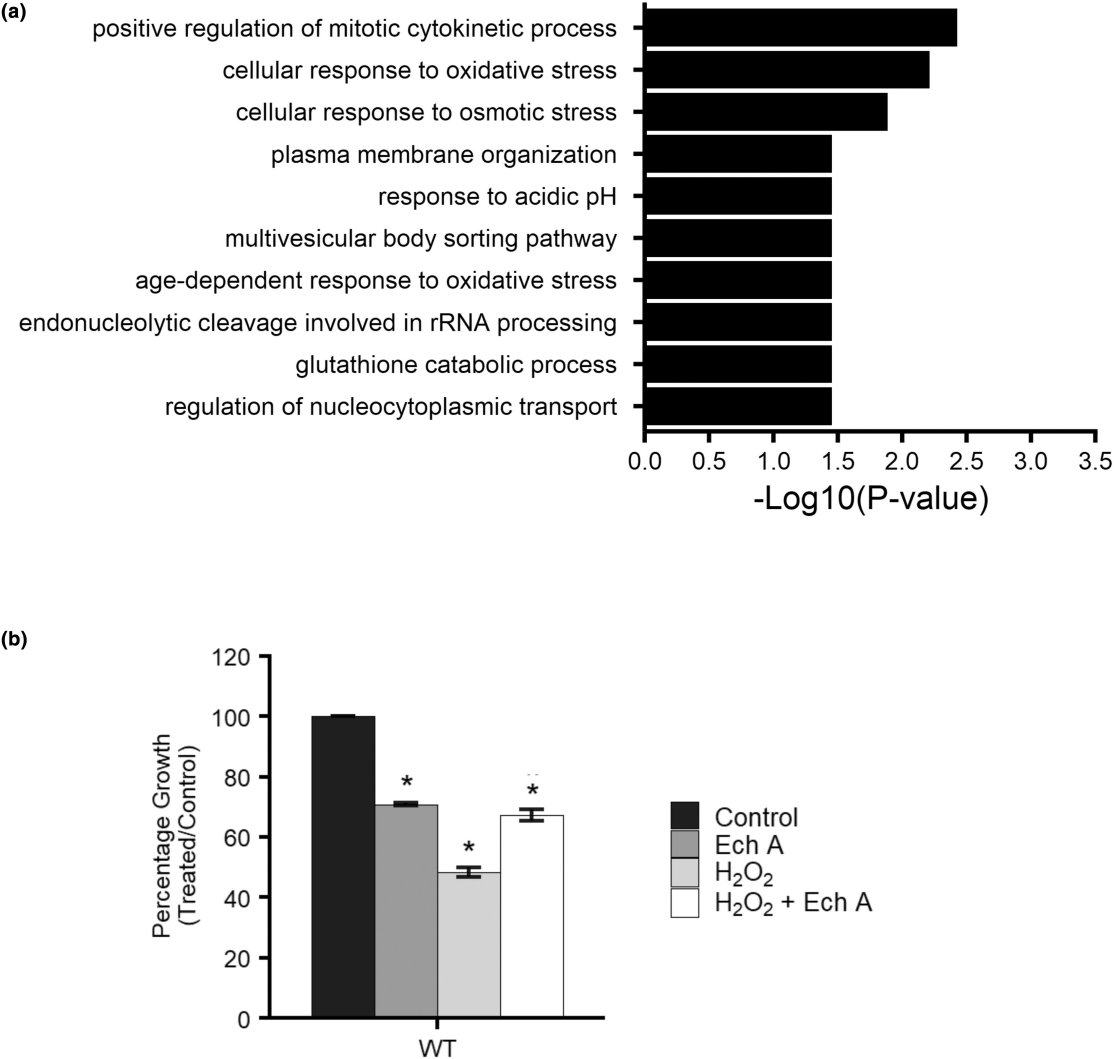
(a) Over‐representation analysis of the 20 Ech A‐sensitive gene deletion mutants reveals enrichment for oxidative stress. Enrichment analysis was conducted using YeastEnrichr. (b) Ech A exhibits antioxidant activity against hydrogen peroxide‐induced oxidative stress. Percentage growth of wildtype grown with and without 1 μg/mL of Ech A and with and without 1.5 mM H_2_O_2_. Data shown as mean of technical triplicates ± *SD*; *p ≤ .05, student's *t*‐test comparing treated to the control.

To determine if there was over‐representation for any particular biological process or molecular function among the 20 genes sensitive to Ech A, enrichment analysis was conducted using YeastEnrichr (Kuleshov et al., 2016). For GO biological process, there was an over‐representation in genes involved in the response to oxidative stress and the response to other stress factors such as heat, salt, and acids (Figure 7a). To experimentally validate the enrichment for oxidative stress, growth of Ech A‐treated yeast was monitored in the presence and absence of a well‐established oxidant hydrogen peroxide (H_2_O_2_), which increased the concentration of reactive oxygen species and induced oxidative stress. The wildtype strain (BY4741) was grown in the presence of control media in the absence or presence of Ech A, H_2_O_2_, or co‐treatment of Ech A + H_2_O_2_ (Figure 7b). While H_2_O_2_ inhibited growth by 50%, growth was improved by 20% when co‐treated with Ech A and H_2_O_2_. These results demonstrate the antioxidant activity of Ech A in the yeast model.

## 4 DISCUSSION

Using *Saccharomyces cerevisiae*, unbiased genome‐wide genomic and proteomic level analysis tools provided further insight into the mode of action of Echinochrome A (Ech A). It was identified that 92 proteins had significant changes in protein abundance caused by Ech A treatment, with an overall enrichment theme of specific changes in DNA replication, repair and RNA binding at 30 min, followed by metabolism of metal ions (specifically iron and copper) from 60–240 min. These results are consistent with the transcriptional response of yeast cells during iron deficiency (Romero et al., 2019). Further analysis indicated that Ech A chelates iron ions, and that supplementation with iron can negate the growth inhibition caused by Ech A. Additionally, it was identified that 20 single gene deletion strains showed high sensitivity to Ech A, which were enriched in genes involved in the cell response to oxidative stress. Our demonstration of antioxidant activity illustrates that, like in mammalian cells, antioxidant properties were present in the yeast model. The underlying genes and proteins in yeast thus provide insight into the molecular mechanisms of iron chelation and antioxidant activity, of which both bioactivities are conserved from yeast to mammalian cells (Forman & Zhang, 2021; Shakoury‐Elizeh et al., 2010) and have each been shown to be involved in the therapeutic efficacy of Ech A in treating a diversity of ailments and diseases (Kim et al., 2021). Given the diversity of these potential applications for Ech A, we propose these fundamental mechanisms observed in the model eukaryotic yeast cell to be involved in the mechanism of many of these applications.

The spectrophotometric spectrum of Ech A is altered when ferrous iron is added, indicating that a formation of an iron‐Ech A complex is possible—although the addition of EDTA did not restore the UV–visible spectrum of Ech A to the original state (Lebedev et al., 2005). Iron is evenly distributed between Ech A and EDTA when ferrous iron is added to a mixture of the two compounds at a molar ratio of 40:100 (Lebedev et al., 2005). These results align with our observation that a lower concentration of Ech A is needed to bind to the same amount of iron ions as EDTA. As the deficiency of cellular iron triggers global changes in translation as organisms are dependent on iron as an essential redox co‐factor in fundamental cellular processes (Romero et al., 2019), our results provide additional insight notably that the protein abundance changes at the 30‐min time point involved in DNA binding, replication and repair and mRNA binding occur during the first 30 min of Ech A treatment. This may mean that these proteins and their orthologues could be primary responders to iron deficiency in yeast and mammalian cells. Tis11 and Rad57 are the only two proteins that had a change in protein abundance which have a reported connection with iron and with one of the enrichments at the 30‐min time point. TIS11 is a mRNA‐binding protein that is expressed during iron starvation or DNA replication stress (Rutherford et al., 2001; Tkach et al., 2012), which would be consistent with our observations that TIS11 protein expression is increased with Ech A treatment that induces DNA replication stress and alters the concentration of available iron ions. RAD57 stabilizes the binding of Rad51 to single‐stranded DNA and is involved in the recombinational repair of double‐strand breaks of DNA during vegetative growth and meiosis (Cherry et al., 2012). Having an increase in the Rad57 protein was less expected. Interestingly, the gene deletion mutant *rad57Δ* has shown increased sensitivity to iron sulphate (Jo et al., 2008). Six other genes that resulted in the DNA replication/repair and RNA binding enrichments at the 30‐min time point include the POL32 subunit of DNA polymerase delta, the CAC2 component of the chromatin assembly complex, the RNA exonuclease REX3, the single‐stranded DNA binding protein SLD2, the QRI7 gene essential for the modification of mitochondrial tRNAs, and the kinetochore protein CNN1. The connection of these genes and iron homeostasis have not yet been reported which may reflect novel links between DNA replication/repair and iron homeostasis.

Our unbiased proteomic and genomic analyses identified a number of mitochondrial genes and proteins involved in the metabolism of Ech A, which is consistent with the targeted investigation of mitochondrial genes and proteins (Jeong, Kim, Song, Noh, et al., 2014). The mitochondrial proteins in yeast that did show a significant change in abundance are involved in metal ion metabolism (specifically iron ions), tRNA wobble and oxidative stress. In addition to altered abundance of mitochondrial proteins, deletion of mitochondrial genes resulted in high sensitivity to Ech A. Notably, the most sensitive mitochondrial gene was the cardiolipin synthase (CRD1). CRD1 is integral to the synthesis of cardiolipin, a key phospholipid species in the mitochondrial membrane that is required for mitochondrial membrane function and particularly sensitive to reactive oxygen species (Malina et al., 2018). Lipid peroxidation is caused during oxidative stress where the free radicals such as hydroxyl radicals attack unsaturated lipids such as cardiolipins, resulting in oxidative damage due to loss of normal function to organelles such as the mitochondria, culminating in cell/tissue death (Ayala et al., 2014). The presence of iron is a key catalyst for lipid peroxidation given the iron chelator deferoxamine inhibited the production of hydroxyl radicals, reduced lipid peroxidation and in turn protected cells against post‐ischemic reperfusion that otherwise damages the heart, lung, kidney, and brain tissues (Menasché et al., 1990). Our results identifying cardiolipin to Ech A bioactivity in yeast suggest cardiolipin may be a part of the therapeutic mechanism already observed in mammalian cells where Ech A reduced ROS levels, reduced lipid peroxidation, and increased mitochondrial function (Jeong, Kim, Song, Lee, et al., 2014; Jeong, Kim, Song, Noh, et al., 2014; Kim et al., 2021; Seo et al., 2015). It is plausible that iron chelation by Ech A can inhibit the activity of the Haber‐Weiss reaction, whereby superoxides (O_2_^•–^) convert Fe^3+^ to Fe^2+^, and inhibit the Fenton reaction catalyzed by the iron ion Fe^2+^ converting H_2_O_2_ into hydroxyl radicals (•OH) (Ayala et al., 2014), resulting in a reduction of hydroxyl radicals being produced and therefore protection from lipid peroxidation.

The Ech A‐treated *ipk1Δ* strain showed one of the highest sensitivities of the 4800 deletion strains with practically no growth compared to untreated cells. IPK1 is an inositol 1,3,4,5,6‐pentakisphosphate 2‐kinase, that is required for the synthesis of phytate that is integral to cell function (Cherry et al., 2012). Interestingly, phytate is a natural metal ion chelator and binds to zinc, iron, and calcium. Phytate is generally found in high concentrations in lower‐income plant‐based diets, resulting in a deficiency of zinc, iron, and calcium (Gibson et al., 2010). The deletion of IPK1 causes dysfunction to the mitochondria via compromised mitochondrial respiratory chain complexes, depolarization of mitochondrial membrane potential, reduced ATP synthesis and mitochondrial structural abnormalities (Zhu et al., 2020). These changes may be the reasons why we see dramatic sensitivity of the *ipk1Δ* strain to Ech A, as the loss of normal mitochondrial function renders the cells more susceptible to stress. Further assays to measure mitochondrial membrane potential and the abundance of cardiolipin will be needed to investigate these phenotypes and provide further insight into the importance of IPK1 and CRD1 in the metabolism of Ech A.

In conclusion, by studying Ech A, we explored the pharmaceutical potential of Ech A as a waste product (as the spine of the kina shell, where Ech A is located, is not eaten with the roe). We used proteomic and genomic analysis to better understand the antioxidant and iron chelating activities of Ech A. As Ech A protects cells from oxidative stress by scavenging free radicals more potently than established antioxidants such as α‐Tocopherol (Lebedev et al., 2005), Ech A may protect cells from free radicals that are already present in the cells, or alternatively, inhibit the production of new free radicals by chelating the iron ions that would normally catalyze the iron‐mediated Fenton reaction. This combination of free radical scavenging and iron chelation potentially makes Ech A a better antioxidant than current established antioxidants. As an iron chelator and antioxidant, Ech A would be predicted to inhibit ferroptosis, a type of programmed cell death dependent on iron homeostasis and lipid peroxidation that is involved in the onset and progression of cancer, Alzheimer's disease, and Parkinson's disease, diabetes, and cardiovascular disease (Jiang et al., 2021). These predictions would be consistent with Ech A preventing post‐menopausal salivary gland dysfunction by a mechanism that includes inhibition of ferroptosis (Kim et al., 2022). Future in vitro and in vivo research in these various disease models can investigate whether inhibition of ferroptosis is a fundamental mechanism of Ech A via the genes and pathways identified in this study.

## AUTHOR CONTRIBUTIONS

**Joseph Hammond:** Conceptualization (equal); data curation (equal); formal analysis (equal); investigation (equal); methodology (equal); validation (equal); visualization (equal); writing – original draft (equal); writing – review and editing (equal). **Isabella M. Das:** Visualization (equal); writing – review and editing (equal). **Ruihana Paenga:** Conceptualization (equal); funding acquisition (equal); project administration (equal); resources (equal). **Manu Caddie:** Conceptualization (equal); funding acquisition (equal); project administration (equal); resources (equal). **Damian Skinner:** Conceptualization (equal); funding acquisition (equal); project administration (equal); resources (equal); writing – review and editing (equal). **Jeffrey P. Sheridan:** Formal analysis (equal); investigation (equal); methodology (equal); visualization (equal); writing – review and editing (equal). **Matthew R. Miller:** Conceptualization (equal); funding acquisition (equal); investigation (equal); methodology (equal); project administration (equal); writing – review and editing (equal). **Andrew B. Munkacsi:** Conceptualization (equal); investigation (equal); methodology (equal); resources (equal); supervision (equal); writing – original draft (equal); writing – review and editing (equal).

## FUNDING INFORMATION

This work was supported by Hikurangi Bioactives Limited Partnership and the Sustainable Seas National Science Challenge program “Huataukīna tō iwi e: Developing marine bioactives from kina”.

## CONFLICT OF INTEREST STATEMENT

The authors declare no competing conflict of interest.

## Supporting information


**Figure S1.** Ech A treatment significantly changes protein abundance of proteins involved in drug response (CIS1, YLR179C) and metal ion metabolism (ARN1, TIS11, SMF3, CCC2, COT1). GFP‐tagged yeast strains were treated with and without 2 μg/mL Ech A and imaged every 30 min over 4 h. Each strain was normalized to the RFP in the control. Line graphs of each strain with the mean arbitrary intensity units (representing GFP fluorescence) of each control and treated replicate over the 4‐h time‐lapse ± *SD*.
**Figure S2.** Ech A treatment significantly changes protein abundance of proteins involved in autophagy (SAM3) and unknown functions (YDR476C, APD1, YCR087C‐A). GFP‐tagged yeast strains were treated with and without 2 μg/mL Ech A and imaged every 30 min over 4 h. Each strain was normalized to the RFP in the control. Line graphs of each strain with the mean arbitrary intensity units (representing GFP fluorescence) of each control and treated replicate over the 4‐h time‐lapse ± *SD*.

**Table S1.** Z‐scores reflecting the ratio of GFP fluorescence in treated and untreated cells of each protein that had a significant change in abundance due to Ech A treatment. The z‐score was calculated every 30 min for 4 h for 4100 strains and only significant changes are shown here.
**Table S2.** Annotation of mitochondrial proteins that were significantly altered in abundance due to Ech A treatment. The open reading frame (ORF), protein name and a brief description of function are provided for each protein. Description of function was obtained from Saccharomyces Genome Database.
**Table S3.** Annotation of proteins involved in drug response that were significantly altered in abundance due to Ech A treatment. gene deletion strains sensitive to Ech A. The open reading frame (ORF), protein name and a brief description of function are provided for each protein. Description of function was obtained from Saccharomyces Genome Database.
**Table S4.** Annotation of proteins involved in metal ion metabolism that were significantly altered in abundance due to Ech A treatment. gene deletion strains sensitive to Ech A. The open reading frame (ORF), protein name and a brief description of function are provided for each protein. Description of function was obtained from Saccharomyces Genome Database.
**Table S5.** Annotation of gene deletion strains sensitive to Ech A. Growth was calculated for 4800 strains and only significant changes are shown here. The open reading frame (ORF), gene name and a brief description of function are provided for each gene. Description of function was obtained from Saccharomyces Genome Database.

## Data Availability

All data reported herein are available in this manuscript.

## References

[fsn34140-bib-0001] Alexander, D. B. , & Zuberer, D. A. (1991). Use of chrome azurol‐S reagents to evaluate siderophore production by rhizosphere bacteria. Biology and Fertility of Soils, 12(1), 39–45. 10.1007/bf00369386

[fsn34140-bib-0002] Artyukov, A. A. , Zelepuga, E. A. , Bogdanovich, L. N. , Lupach, N. M. , Novikov, V. L. , Rutckova, T. A. , & Kozlovskaya, E. P. (2020). Marine polyhydroxynaphthoquinone, Echinochrome A: Prevention of atherosclerotic inflammation and probable molecular targets. Journal of Clinical Medicine, 9(5), 1494. 10.3390/jcm9051494 32429179 PMC7291202

[fsn34140-bib-0003] Ayala, A. , Munoz, M. F. , & Arguelles, S. (2014). Lipid peroxidation: Production, metabolism, and signaling mechanisms of malondialdehyde and 4‐hydroxy‐2‐nonenal. Oxidative Medicine and Cellular Longevity, 2014, 360438. 10.1155/2014/360438 24999379 PMC4066722

[fsn34140-bib-0004] Bircham, P. W. , Maass, D. R. , Roberts, C. A. , Kiew, P. Y. , Low, Y. S. , Yegambaram, M. , Matthews, J. , Jack, C. A. , & Atkinson, P. H. (2011). Secretory pathway genes assessed by high‐throughput microscopy and synthetic genetic array analysis. Molecular BioSystems, 7(9), 2589–2598. 10.1039/c1mb05175j 21731954

[fsn34140-bib-0005] Carroll, A. R. , Copp, B. R. , Davis, R. A. , Keyzers, R. A. , & Prinsep, M. R. (2023). Marine natural products. Natural Product Reports, 40(2), 275–325. 10.1039/d2np00083k 36786022

[fsn34140-bib-0006] Cherry, J. M. , Hong, E. L. , Amundsen, C. , Balakrishnan, R. , Binkley, G. , Chan, E. T. , Christie, K. R. , Costanzo, M. C. , Dwight, S. S. , Engel, S. R. , Fisk, D. G. , Hirschman, J. E. , Hitz, B. C. , Karra, K. , Krieger, C. J. , Miyasato, S. R. , Nash, R. S. , Park, J. , Skrzypek, M. S. , … Wong, E. D. (2012). *Saccharomyces* Genome Database: The genomics resource of budding yeast. Nucleic Acids Research, 40, D700–D705. 10.1093/nar/gkr1029 22110037 PMC3245034

[fsn34140-bib-0007] Choi, M. R. , Lee, H. , Kim, H. K. , Han, J. , Seol, J. E. , Vasileva, E. A. , Mishchenko, N. P. , Fedoreyev, S. A. , Stonik, V. A. , Ju, W. S. , Kim, D. J. , & Lee, S. R. (2022). Echinochrome A inhibits melanogenesis in B16F10 cells by downregulating CREB signaling. Marine Drugs, 20(9), 555. 10.3390/md20090555 36135744 PMC9502928

[fsn34140-bib-0008] Chong, Y. T. , Koh, J. L. , Friesen, H. , Duffy, S. K. , Cox, M. J. , Moses, A. , Moffat, J. , Boone, C. , & Andrews, B. J. (2015). Yeast proteome dynamics from single cell imaging and automated analysis. Cell, 161(6), 1413–1424. 10.1016/j.cell.2015.04.051 26046442

[fsn34140-bib-0009] Cui, H. , Liu, J. , Vasileva, E. A. , Mishchenko, N. P. , Fedoreyev, S. A. , Stonik, V. A. , & Zhang, Y. (2022). Echinochrome A reverses kidney abnormality and reduces blood pressure in a rat model of preeclampsia. Marine Drugs, 20(11), 722. 10.3390/md20110722 36421999 PMC9699499

[fsn34140-bib-0010] Ekimova, I. V. , Plaksina, D. V. , Pastukhov, Y. F. , Lapshina, K. V. , Lazarev, V. F. , Mikhaylova, E. R. , Polonik, S. G. , Pani, B. , Margulis, B. A. , Guzhova, I. V. , & Nudler, E. (2018). New HSF1 inducer as a therapeutic agent in a rodent model of Parkinson's disease. Experimental Neurology, 306, 199–208. 10.1016/j.expneurol.2018.04.012 29704482

[fsn34140-bib-0011] Fedoreyev, S. A. , Krylova, N. V. , Mishchenko, N. P. , Vasileva, E. A. , Pislyagin, E. A. , Iunikhina, O. V. , Lavrov, V. F. , Svitich, O. A. , Ebralidze, L. K. , & Leonova, G. N. (2018). Antiviral and antioxidant properties of Echinochrome A. Marine Drugs, 16(12), 509. 10.3390/md16120509 30558297 PMC6315383

[fsn34140-bib-0012] Fisheries Infosite: Catch analysis of kina in New Zealand . (2022). Fisheries New Zealand. Retrieved May 19, 2023, from https://fs.fish.govt.nz/Page.aspx?pk=7&tk=100&sc=SUR

[fsn34140-bib-0013] Forman, H. J. , & Zhang, H. (2021). Targeting oxidative stress in disease: Promise and limitations of antioxidant therapy. Nature Reviews. Drug Discovery, 20(9), 689–709. 10.1038/s41573-021-00233-1 34194012 PMC8243062

[fsn34140-bib-0014] Gibson, R. S. , Bailey, K. B. , Gibbs, M. , & Ferguson, E. L. (2010). A review of phytate, iron, zinc, and calcium concentrations in plant‐based complementary foods used in low‐income countries and implications for bioavailability. Food and Nutrition Bulletin, 31(2 Suppl), S134–S146. 10.1177/15648265100312s206 20715598

[fsn34140-bib-0015] Haefner, B. (2003). Drugs from the deep: Marine natural products as drug candidates. Drug Discovery Today, 8(12), 536–544. 10.1016/s1359-6446(03)02713-2 12821301

[fsn34140-bib-0016] Hillenmeyer, M. E. , Fung, E. , Wildenhain, J. , Pierce, S. E. , Hoon, S. , Lee, W. , Proctor, M. , St Onge, R. P. , Tyers, M. , Koller, D. , Altman, R. B. , Davis, R. W. , Nislow, C. , & Giaever, G. (2008). The chemical genomic portrait of yeast: Uncovering a phenotype for all genes. Science, 320(5874), 362–365. 10.1126/science.1150021 18420932 PMC2794835

[fsn34140-bib-0017] Hou, Y. , Carne, A. , McConnell, M. , Mros, S. , Vasileva, E. A. , Mishchenko, N. P. , Burrow, K. , Wang, K. , Bekhit, A. A. , & Bekhit, A. E. A. (2020). PHNQ from *Evechinus chloroticus* sea urchin supplemented with calcium promotes mineralization in Saos‐2 human bone cell line. Marine Drugs, 18(7), 373. 10.3390/md18070373 32707634 PMC7404214

[fsn34140-bib-0018] Hou, Y. , Vasileva, E. A. , Mishchenko, N. P. , Carne, A. , McConnell, M. , & Bekhit, A. E. A. (2019). Extraction, structural characterization and stability of polyhydroxylated naphthoquinones from shell and spine of New Zealand sea urchin (*Evechinus chloroticus*). Food Chemistry, 272, 379–387. 10.1016/j.foodchem.2018.08.046 30309558

[fsn34140-bib-0019] Huh, W. K. , Falvo, J. V. , Gerke, L. C. , Carroll, A. S. , Howson, R. W. , Weissman, J. S. , & O'Shea, E. K. (2003). Global analysis of protein localization in budding yeast. Nature, 425(6959), 686–691. 10.1038/nature02026 14562095

[fsn34140-bib-0020] James, J. , Fiji, N. , Roy, D. , Andrew Mg, D. , Shihabudeen, M. S. , Chattopadhyay, D. , & Thirumurugan, K. (2015). A rapid method to assess reactive oxygen species in yeast using H2DCF‐DA. Analytical Methods, 7(20), 8572–8575. 10.1039/c5ay02278a

[fsn34140-bib-0021] Jeong, S. H. , Kim, H. K. , Song, I. S. , Lee, S. J. , Ko, K. S. , Rhee, B. D. , Kim, N. , Mishchenko, N. P. , Fedoryev, S. A. , Stonik, V. A. , & Han, J. (2014). Echinochrome A protects mitochondrial function in cardiomyocytes against cardiotoxic drugs. Marine Drugs, 12(5), 2922–2936. 10.3390/md12052922 24828295 PMC4052324

[fsn34140-bib-0022] Jeong, S. H. , Kim, H. K. , Song, I. S. , Noh, S. J. , Marquez, J. , Ko, K. S. , Rhee, B. D. , Kim, N. , Mishchenko, N. P. , Fedoreyev, S. A. , Stonik, V. A. , & Han, J. (2014). Echinochrome A increases mitochondrial mass and function by modulating mitochondrial biogenesis regulatory genes. Marine Drugs, 12(8), 4602–4615. 10.3390/md12084602 25196935 PMC4145333

[fsn34140-bib-0023] Jiang, X. , Stockwell, B. R. , & Conrad, M. (2021). Ferroptosis: Mechanisms, biology and role in disease. Nature Reviews. Molecular Cell Biology, 22(4), 266–282. 10.1038/s41580-020-00324-8 33495651 PMC8142022

[fsn34140-bib-0024] Jo, W. J. , Loguinov, A. , Chang, M. , Wintz, H. , Nislow, C. , Arkin, A. P. , Giaever, G. , & Vulpe, C. D. (2008). Identification of genes involved in the toxic response of *Saccharomyces cerevisiae* against iron and copper overload by parallel analysis of deletion mutants. Toxicological Sciences, 101(1), 140–151. 10.1093/toxsci/kfm226 17785683

[fsn34140-bib-0025] Kim, H. K. , Vasileva, E. A. , Mishchenko, N. P. , Fedoreyev, S. A. , & Han, J. (2021). Multifaceted clinical effects of Echinochrome. Marine Drugs, 19(8), 412. 10.3390/md19080412 34436251 PMC8400489

[fsn34140-bib-0026] Kim, H. K. , Youm, J. B. , Jeong, S. H. , Lee, S. R. , Song, I. S. , Ko, T. H. , Pronto, J. R. , Ko, K. S. , Rhee, B. D. , Kim, N. , Nilius, B. , Mischchenko, N. P. , Fedoreyev, S. A. , Stonik, V. A. , & Han, J. (2015). Echinochrome A regulates phosphorylation of phospholamban Ser16 and Thr17 suppressing cardiac SERCA2A Ca^2+^ reuptake. Pflügers Archiv, 467(10), 2151–2163. 10.1007/s00424-014-1648-2 25410495

[fsn34140-bib-0027] Kim, J. M. , Shin, S. C. , Cheon, Y. I. , Kim, H. S. , Park, G. C. , Kim, H. K. , Han, J. , Seol, J. E. , Vasileva, E. A. , Mishchenko, N. P. , Fedoreyev, S. A. , Stonik, V. A. , & Lee, B. J. (2022). Effect of Echinochrome A on submandibular gland dysfunction in ovariectomized rats. Marine Drugs, 20(12), 729. 10.3390/md20120729 36547876 PMC9785380

[fsn34140-bib-0028] Kim, R. , Hur, D. , Kim, H. K. , Han, J. , Mishchenko, N. P. , Fedoreyev, S. A. , Stonik, V. A. , & Chang, W. (2019). Echinochrome A attenuates cerebral ischemic injury through regulation of cell survival after middle cerebral artery occlusion in rat. Marine Drugs, 17(9), 501. 10.3390/md17090501 31466244 PMC6780833

[fsn34140-bib-0029] Kim, S. E. , Chung, E. D. S. , Vasileva, E. A. , Mishchenko, N. P. , Fedoreyev, S. A. , Stonik, V. A. , Kim, H. K. , Nam, J. H. , & Kim, S. J. (2023). Multiple effects of Echinochrome A on selected ion channels implicated in skin physiology. Marine Drugs, 21(2), 78. 10.3390/md21020078 36827119 PMC9963876

[fsn34140-bib-0030] Kraus, O. Z. , Grys, B. T. , Ba, J. , Chong, Y. , Frey, B. J. , Boone, C. , & Andrews, B. J. (2017). Automated analysis of high‐content microscopy data with deep learning. Molecular Systems Biology, 13(4), 924. 10.15252/msb.20177551 28420678 PMC5408780

[fsn34140-bib-0031] Kuleshov, M. V. , Jones, M. R. , Rouillard, A. D. , Fernandez, N. F. , Duan, Q. , Wang, Z. , Koplev, S. , Jenkins, S. L. , Jagodnik, K. M. , Lachmann, A. , McDermott, M. G. , Monteiro, C. D. , Gundersen, G. W. , & Ma'ayan, A. (2016). Enrichr: A comprehensive gene set enrichment analysis web server 2016 update. Nucleic Acids Research, 44(W1), W90–W97. 10.1093/nar/gkw377 27141961 PMC4987924

[fsn34140-bib-0032] Lazarev, V. F. , Nikotina, A. D. , Mikhaylova, E. R. , Nudler, E. , Polonik, S. G. , Guzhova, I. V. , & Margulis, B. A. (2016). Hsp70 chaperone rescues C6 rat glioblastoma cells from oxidative stress by sequestration of aggregating GAPDH. Biochemical and Biophysical Research Communications, 470(3), 766–771. 10.1016/j.bbrc.2015.12.076 26713364

[fsn34140-bib-0033] Lebedev, A. V. , Ivanova, M. V. , & Levitsky, D. O. (2005). Echinochrome, a naturally occurring iron chelator and free radical scavenger in artificial and natural membrane systems. Life Sciences, 76(8), 863–875. 10.1016/j.lfs.2004.10.007 15589964

[fsn34140-bib-0034] Lebed'ko, O. A. , Ryzhavskii, B. Y. , & Demidova, O. V. (2015). Effect of antioxidant Echinochrome A on bleomycin‐induced pulmonary fibrosis. Bulletin of Experimental Biology and Medicine, 159(3), 351–354. 10.1007/s10517-015-2960-3 26201908

[fsn34140-bib-0035] Lee, A. Y. , St Onge, R. P. , Proctor, M. J. , Wallace, I. M. , Nile, A. H. , Spagnuolo, P. A. , Jitkova, Y. , Gronda, M. , Wu, Y. , Kim, M. K. , Cheung‐Ong, K. , Torres, N. P. , Spear, E. D. , Han, M. K. , Schlecht, U. , Suresh, S. , Duby, G. , Heisler, L. E. , Surendra, A. , … Giaever, G. (2014). Mapping the cellular response to small molecules using chemogenomic fitness signatures. Science, 344(6180), 208–211. 10.1126/science.1250217 24723613 PMC4254748

[fsn34140-bib-0036] Lee, S. R. , Pronto, J. R. , Sarankhuu, B. E. , Ko, K. S. , Rhee, B. D. , Kim, N. , Mishchenko, N. P. , Fedoreyev, S. A. , Stonik, V. A. , & Han, J. (2014). Acetylcholinesterase inhibitory activity of pigment echinochrome A from sea urchin *Scaphechinus mirabilis* . Marine Drugs, 12(6), 3560–3573. 10.3390/md12063560 24918454 PMC4071590

[fsn34140-bib-0037] Lennikov, A. , Kitaichi, N. , Noda, K. , Mizuuchi, K. , Ando, R. , Dong, Z. , Fukuhara, J. , Kinoshita, S. , Namba, K. , Ohno, S. , & Ishida, S. (2014). Amelioration of endotoxin‐induced uveitis treated with the sea urchin pigment echinochrome in rats. Molecular Vision, 20, 171–177. 24520186 PMC3919668

[fsn34140-bib-0038] Malina, C. , Larsson, C. , & Nielsen, J. (2018). Yeast mitochondria: An overview of mitochondrial biology and the potential of mitochondrial systems biology. FEMS Yeast Research, 18(5), foy040. 10.1093/femsyr/foy040 29788060

[fsn34140-bib-0039] McQuin, C. , Goodman, A. , Chernyshev, V. , Kamentsky, L. , Cimini, B. A. , Karhohs, K. W. , Doan, M. , Ding, L. , Rafelski, S. M. , Thirstrup, D. , Wiegraebe, W. , Singh, S. , Becker, T. , Caicedo, J. C. , & Carpenter, A. E. (2018). CellProfiler 3.0: Next‐generation image processing for biology. PLoS Biology, 16(7), e2005970. 10.1371/journal.pbio.2005970 29969450 PMC6029841

[fsn34140-bib-0040] Menasché, P. , Antebi, H. , Alcindor, L. G. , Teiger, E. , Perez, G. , Giudicelli, Y. , Nordmann, R. , & Piwnica, A. (1990). Iron chelation by deferoxamine inhibits lipid peroxidation during cardiopulmonary bypass in humans. Circulation, 82(5 Suppl), Iv390–Iv396. 2225430

[fsn34140-bib-0041] Mishchenko, N. P. , Krylova, N. V. , Iunikhina, O. V. , Vasileva, E. A. , Likhatskaya, G. N. , Pislyagin, E. A. , Tarbeeva, D. V. , Dmitrenok, P. S. , & Fedoreyev, S. A. (2020). Antiviral potential of sea urchin aminated spinochromes against Herpes Simplex Virus Type 1. Marine Drugs, 18(11), 550. 10.3390/md18110550 33167501 PMC7694471

[fsn34140-bib-0042] Mohamed, A. S. (2021). Echinochrome exhibits antitumor activity against Ehrlich ascites carcinoma in swiss albino mice. Nutrition and Cancer, 73(1), 124–132. 10.1080/01635581.2020.1737152 32151164

[fsn34140-bib-0043] Mohamed, A. S. , Soliman, A. M. , & Marie, M. A. S. (2016). Mechanisms of echinochrome potency in modulating diabetic complications in liver. Life Sciences, 151, 41–49. 10.1016/j.lfs.2016.03.007 26947587

[fsn34140-bib-0044] Molimau‐Samasoni, S. , Woolner, V. H. , Foliga, S. T. , Robichon, K. , Patel, V. , Andreassend, S. K. , Sheridan, J. P. , Te Kawa, T. , Gresham, D. , Miller, D. , Sinclair, D. J. , La Flamme, A. C. , Melnik, A. V. , Aron, A. , Dorrestein, P. C. , Atkinson, P. H. , Keyzers, R. A. , & Munkacsi, A. B. (2021). Functional genomics and metabolomics advance the ethnobotany of the Samoan traditional medicine “matalafi”. Proceedings of the National Academy of Sciences of the United States of America, 118(45), e2100880118. 10.1073/pnas.2100880118 34725148 PMC8609454

[fsn34140-bib-0045] Newman, D. J. , & Cragg, G. M. (2020). Natural products as sources of new drugs over the nearly four decades from 01/1981 to 09/2019. Journal of Natural Products, 83(3), 770–803. 10.1021/acs.jnatprod.9b01285 32162523

[fsn34140-bib-0046] Ogawa, T. , Tsubakiyama, R. , Kanai, M. , Koyama, T. , Fujii, T. , Iefuji, H. , Soga, T. , Kume, K. , Miyakawa, T. , Hirata, D. , & Mizunuma, M. (2016). Stimulating S‐adenosyl‐l‐methionine synthesis extends lifespan via activation of AMPK. Proceedings of the National Academy of Sciences of the United States of America, 113(42), 11913–11918. 10.1073/pnas.1604047113 27698120 PMC5081624

[fsn34140-bib-0047] Park, G. T. , Yoon, J. W. , Yoo, S. B. , Song, Y. C. , Song, P. , Kim, H. K. , Han, J. , Bae, S. J. , Ha, K. T. , Mishchenko, N. P. , Fedoreyev, S. A. , Stonik, V. A. , Kim, M. B. , & Kim, J. H. (2021). Echinochrome A treatment alleviates fibrosis and inflammation in bleomycin‐induced scleroderma. Marine Drugs, 19(5), 237. 10.3390/md19050237 33922418 PMC8146844

[fsn34140-bib-0048] Parsons, A. B. , Lopez, A. , Givoni, I. E. , Williams, D. E. , Gray, C. A. , Porter, J. , Chua, G. , Sopko, R. , Brost, R. L. , Ho, C. H. , Wang, J. , Ketela, T. , Brenner, C. , Brill, J. A. , Fernandez, G. E. , Lorenz, T. C. , Payne, G. S. , Ishihara, S. , Ohya, Y. , … Boone, C. (2006). Exploring the mode‐of‐action of bioactive compounds by chemical‐genetic profiling in yeast. Cell, 126(3), 611–625. 10.1016/j.cell.2006.06.040 16901791

[fsn34140-bib-0049] Pham, T. K. , Nguyen, T. H. T. , Yun, H. R. , Vasileva, E. A. , Mishchenko, N. P. , Fedoreyev, S. A. , Stonik, V. A. , Vu, T. T. , Nguyen, H. Q. , Cho, S. W. , Kim, H. K. , & Han, J. (2023). Echinochrome A prevents diabetic nephropathy by inhibiting the PKC‐Iota pathway and enhancing renal mitochondrial function in db/db mice. Marine Drugs, 21(4), 222. 10.3390/md21040222 37103361 PMC10142928

[fsn34140-bib-0050] Romero, A. M. , Ramos‐Alonso, L. , Montella‐Manuel, S. , Garcia‐Martinez, J. , de la Torre‐Ruiz, M. A. , Perez‐Ortin, J. E. , Martinez‐Pastor, M. T. , & Puig, S. (2019). A genome‐wide transcriptional study reveals that iron deficiency inhibits the yeast TORC1 pathway. Biochimica et Biophysica Acta, Gene Regulatory Mechanisms, 1862(9), 194414. 10.1016/j.bbagrm.2019.194414 31394264

[fsn34140-bib-0051] Rubilar, T. , Barbieri, E. S. , Gazquez, A. , & Avaro, M. (2021). Sea urchin pigments: Echinochrome A and its potential implication in the cytokine storm syndrome. Marine Drugs, 19(5), 267. 10.3390/md19050267 34064550 PMC8151293

[fsn34140-bib-0052] Rutherford, J. C. , Jaron, S. , Ray, E. , Brown, P. O. , & Winge, D. R. (2001). A second iron‐regulatory system in yeast independent of Aft1p. Proceedings of the National Academy of Sciences of the United States of America, 98(25), 14322–14327. 10.1073/pnas.261381198 11734641 PMC64680

[fsn34140-bib-0053] Sadek, S. A. , Hassanein, S. S. , Mohamed, A. S. , Soliman, A. M. , & Fahmy, S. R. (2021). Echinochrome pigment extracted from sea urchin suppress the bacterial activity, inflammation, nociception, and oxidative stress resulted in the inhibition of renal injury in septic rats. Journal of Food Biochemistry, 46(3), e13729. 10.1111/jfbc.13729 33871886

[fsn34140-bib-0054] Schindelin, J. , Arganda‐Carreras, I. , Frise, E. , Kaynig, V. , Longair, M. , Pietzsch, T. , Preibisch, S. , Rueden, C. , Saalfeld, S. , Schmid, B. , Tinevez, J. Y. , White, D. J. , Hartenstein, V. , Eliceiri, K. , Tomancak, P. , & Cardona, A. (2012). Fiji: An open‐source platform for biological‐image analysis. Nature Methods, 9(7), 676–682. 10.1038/nmeth.2019 22743772 PMC3855844

[fsn34140-bib-0055] Sedova, K. , Bernikova, O. , Azarov, J. , Shmakov, D. , Vityazev, V. , & Kharin, S. (2015). Effects of echinochrome on ventricular repolarization in acute ischemia. Journal of Electrocardiology, 48(2), 181–186. 10.1016/j.jelectrocard.2015.01.003 25598075

[fsn34140-bib-0056] Seo, D. Y. , McGregor, R. A. , Noh, S. J. , Choi, S. J. , Mishchenko, N. P. , Fedoreyev, S. A. , Stonik, V. A. , & Han, J. (2015). Echinochrome A improves exercise capacity during short‐term endurance training in rats. Marine Drugs, 13(9), 5722–5731. 10.3390/md13095722 26371013 PMC4584350

[fsn34140-bib-0057] Seol, J. E. , Ahn, S. W. , Seol, B. , Yun, H. R. , Park, N. , Kim, H. K. , Vasileva, E. A. , Mishchenko, N. P. , Fedoreyev, S. A. , Stonik, V. A. , & Han, J. (2021). Echinochrome A protects against ultraviolet B‐induced photoaging by lowering collagen degradation and inflammatory cell infiltration in hairless mice. Marine Drugs, 19(10), 550. 10.3390/md19100550 34677449 PMC8537837

[fsn34140-bib-0058] Shakoury‐Elizeh, M. , Protchenko, O. , Berger, A. , Cox, J. , Gable, K. , Dunn, T. M. , Prinz, W. A. , Bard, M. , & Philpott, C. C. (2010). Metabolic response to iron deficiency in *Saccharomyces cerevisiae* . The Journal of Biological Chemistry, 285(19), 14823–14833. 10.1074/jbc.M109.091710 20231268 PMC2863190

[fsn34140-bib-0059] Song, B. W. , Kim, S. , Kim, R. , Jeong, S. , Moon, H. , Kim, H. , Vasileva, E. A. , Mishchenko, N. P. , Fedoreyev, S. A. , Stonik, V. A. , Lee, M. Y. , Kim, J. , Kim, H. K. , Han, J. , & Chang, W. (2022). Regulation of inflammation‐mediated endothelial to mesenchymal transition with Echinochrome A for improving myocardial dysfunction. Marine Drugs, 20(12), 756. 10.3390/md20120756 36547903 PMC9781361

[fsn34140-bib-0060] Stegmaier, K. , Blinn, C. M. , Bechtel, D. F. , Greth, C. , Auerbach, H. , Muller, C. S. , Jakob, V. , Reijerse, E. J. , Netz, D. J. A. , Schunemann, V. , & Pierik, A. J. (2019). Apd1 and Aim32 are prototypes of bishistidinyl‐coordinated non‐Rieske [2Fe‐2S] proteins. Journal of the American Chemical Society, 141(14), 5753–5765. 10.1021/jacs.8b13274 30879301

[fsn34140-bib-0061] Tang, X. , Nishimura, A. , Ariyoshi, K. , Nishiyama, K. , Kato, Y. , Vasileva, E. A. , Mishchenko, N. P. , Fedoreyev, S. A. , Stonik, V. A. , Kim, H. K. , Han, J. , Kanda, Y. , Umezawa, K. , Urano, Y. , Akaike, T. , & Nishida, M. (2023). Echinochrome prevents sulfide catabolism‐associated chronic heart failure after myocardial infarction in mice. Marine Drugs, 21(1), 52. 10.3390/md21010052 36662225 PMC9863521

[fsn34140-bib-0062] Tkach, J. M. , Yimit, A. , Lee, A. Y. , Riffle, M. , Costanzo, M. , Jaschob, D. , Hendry, J. A. , Ou, J. , Moffat, J. , Boone, C. , Davis, T. N. , Nislow, C. , & Brown, G. W. (2012). Dissecting DNA damage response pathways by analysing protein localization and abundance changes during DNA replication stress. Nature Cell Biology, 14(9), 966–976. 10.1038/ncb2549 22842922 PMC3434236

[fsn34140-bib-0063] Wagih, O. , Usaj, M. , Baryshnikova, A. , VanderSluis, B. , Kuzmin, E. , Costanzo, M. , Myers, C. L. , Andrews, B. J. , Boone, C. M. , & Parts, L. (2013). SGAtools: One‐stop analysis and visualization of array‐based genetic interaction screens. Nucleic Acids Research, 41, W591–W596. 10.1093/nar/gkt400 23677617 PMC3692131

[fsn34140-bib-0064] Yun, H. R. , Ahn, S. W. , Seol, B. , Vasileva, E. A. , Mishchenko, N. P. , Fedoreyev, S. A. , Stonik, V. A. , Han, J. , Ko, K. S. , Rhee, B. D. , Seol, J. E. , & Kim, H. K. (2021). Echinochrome A treatment alleviates atopic dermatitis‐like skin lesions in NC/Nga mice via IL‐4 and IL‐13 suppression. Marine Drugs, 19(11), 622. 10.3390/md19110622 34822493 PMC8625509

[fsn34140-bib-0065] Zhang, F. , Zhao, M. , Braun, D. R. , Ericksen, S. S. , Piotrowski, J. S. , Nelson, J. , Peng, J. , Ananiev, G. E. , Chanana, S. , Barns, K. , Fossen, J. , Sanchez, H. , Chevrette, M. G. , Guzei, I. A. , Zhao, C. , Guo, L. , Tang, W. , Currie, C. R. , Rajski, S. R. , … Bugni, T. S. (2020). A marine microbiome antifungal targets urgent‐threat drug‐resistant fungi. Science, 370(6519), 974–978. 10.1126/science.abd6919 33214279 PMC7756952

[fsn34140-bib-0066] Zhu, H. , Zhu, N. , Peng, L. , Zhang, B. , Yu, Q. , & Li, M. (2020). The inositol polyphosphate kinase Ipk1 transcriptionally regulates mitochondrial functions in *Candida albicans* . FEMS Yeast Research, 20(6), foaa050. 10.1093/femsyr/foaa050 32833009

