# Supplementary figures and images for: Multi‐omic analysis reveals genes and proteins integral to bioactivity of Echinochrome A isolated from the waste stream of the sea urchin industry in Aotearoa New Zealand
Source: Food Sci Nutr. 2024 Apr 2;12(7):4927–43. doi: 10.1002/fsn3.4140 (PMC11266889; doi:10.1002/fsn3.4140)

Figure S1

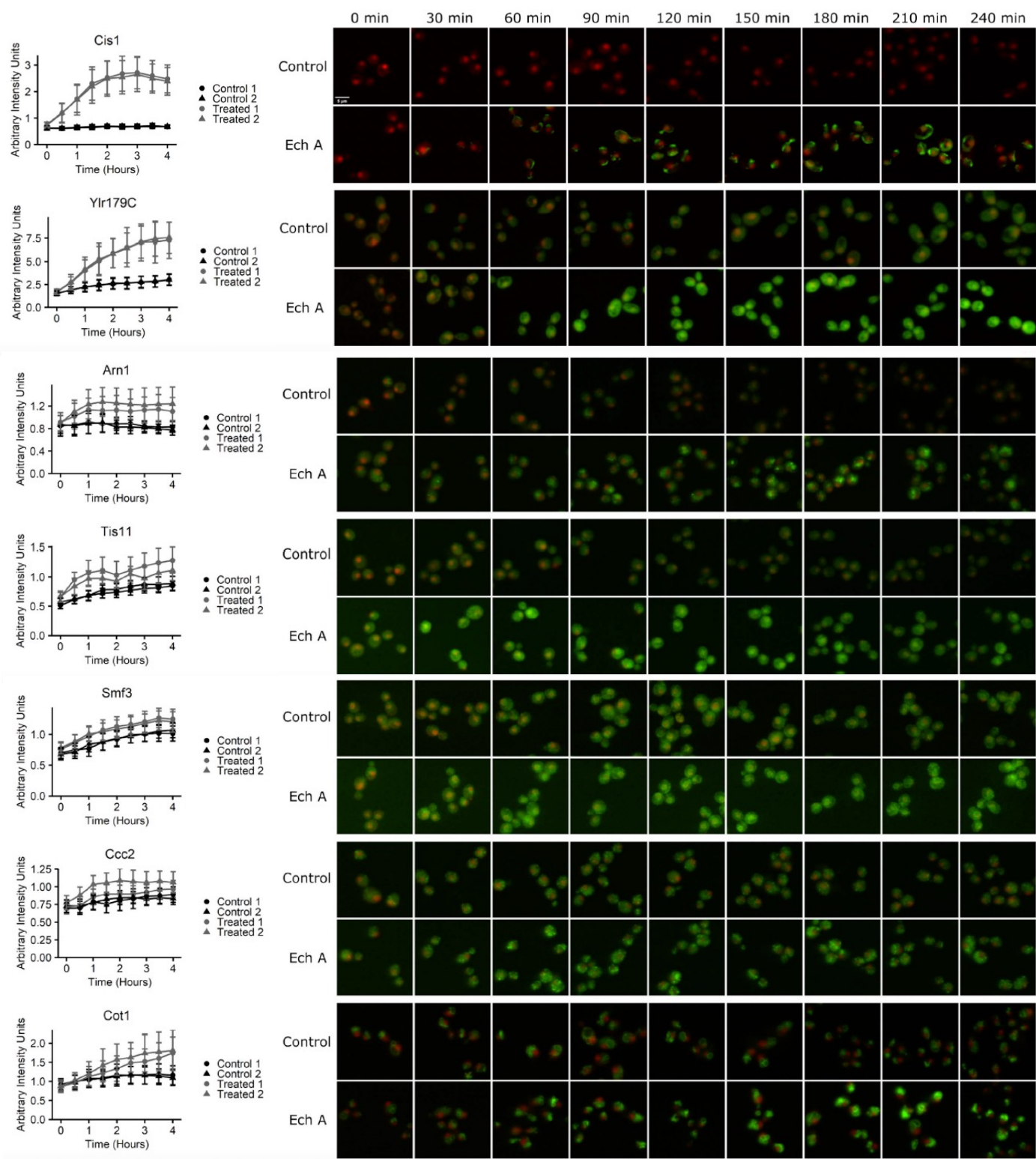

Figure S2

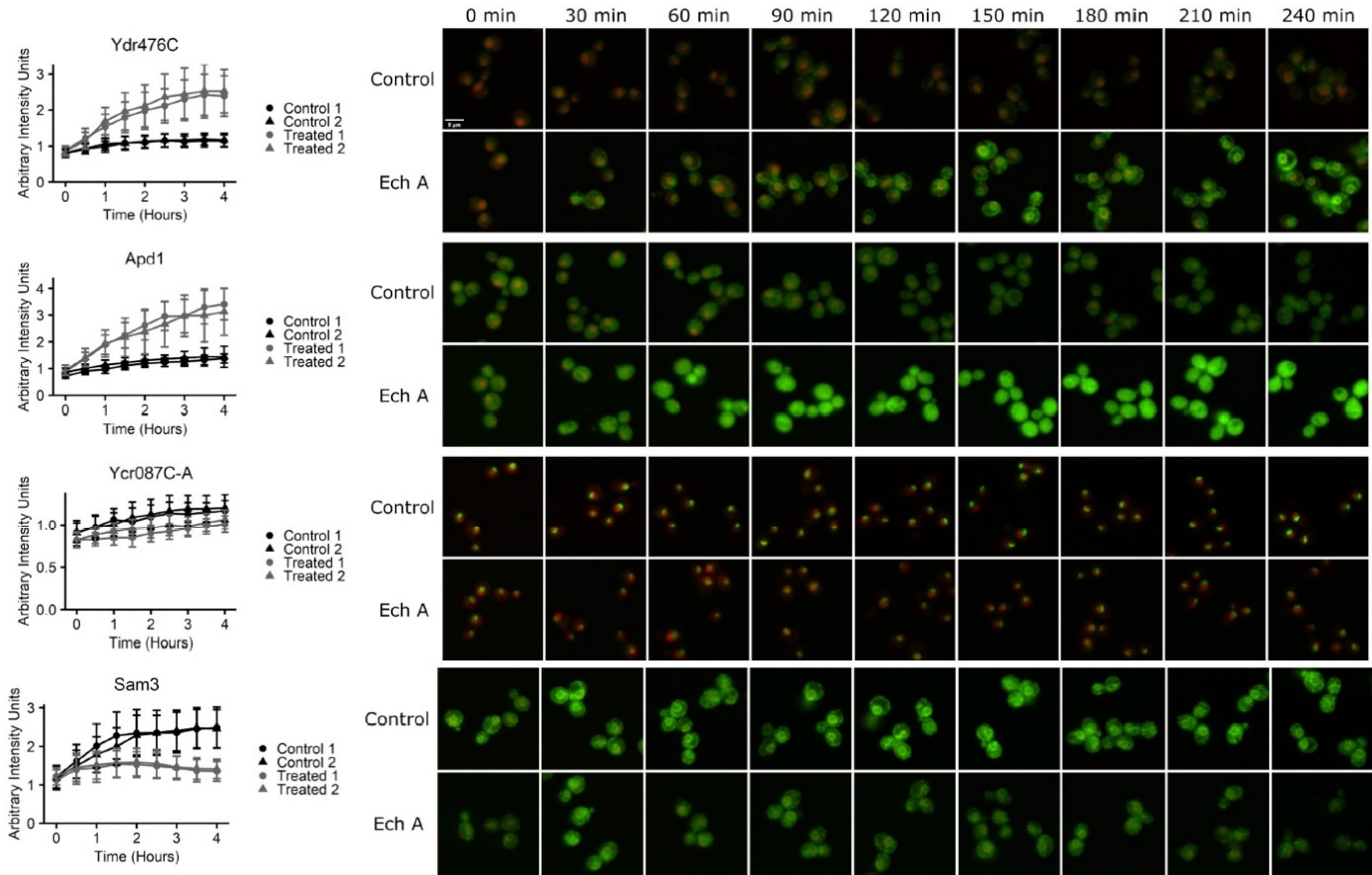

Supplement: Supplementary file 1 — Figure S1. Ech A treatment significantly changes protein abundance of proteins involved in drug response (CIS1, YLR179C) and metal ion metabolism (ARN1, TIS11, SMF3, CCC2, COT1). GFP‐tagged yeast strains were treated with and without 2 μg/mL Ech A and imaged every 30 min over 4 h. Each strain was normalized to the RFP in the control. Line graphs of each strain with the mean arbitrary intensity units (representing GFP fluorescence) of each control and treated replicate over the 4‐h time‐lapse ± SD. Figure S2. Ech A treatment significantly changes protein abundance of proteins involved in autophagy (SAM3) and unknown functions (YDR476C, APD1, YCR087C‐A). GFP‐tagged yeast strains were treated with and without 2 μg/mL Ech A and imaged every 30 min over 4 h. Each strain was normalized to the RFP in the control. Line graphs of each strain with the mean arbitrary intensity units (representing GFP fluorescence) of each control and treated replicate over the 4‐h time‐lapse ± SD. [file FSN3-12-4927-s001.pdf]
