# Supplementary material for: Multi‐omic analysis reveals genes and proteins integral to bioactivity of Echinochrome A isolated from the waste stream of the sea urchin industry in Aotearoa New Zealand
Source: Food Sci Nutr. 2024 Apr 2;12(7):4927–43. doi: 10.1002/fsn3.4140 (PMC11266889; doi:10.1002/fsn3.4140)
Supplement: Supplementary file 2 — Table S1. Z‐scores reflecting the ratio of GFP fluorescence in treated and untreated cells of each protein that had a significant change in abundance due to Ech A treatment. The z‐score was calculated every 30 min for 4 h for 4100 strains and only significant changes are shown here. Table S2. Annotation of mitochondrial proteins that were significantly altered in abundance due to Ech A treatment. The open reading frame (ORF), protein name and a brief description of function are provided for each protein. Description of function was obtained from Saccharomyces Genome Database. Table S3. Annotation of proteins involved in drug response that were significantly altered in abundance due to Ech A treatment. gene deletion strains sensitive to Ech A. The open reading frame (ORF), protein name and a brief description of function are provided for each protein. Description of function was obtained from Saccharomyces Genome Database. Table S4. Annotation of proteins involved in metal ion metabolism that were significantly altered in abundance due to Ech A treatment. gene deletion strains sensitive to Ech A. The open reading frame (ORF), protein name and a brief description of function are provided for each protein. Description of function was obtained from Saccharomyces Genome Database. Table S5. Annotation of gene deletion strains sensitive to Ech A. Growth was calculated for 4800 strains and only significant changes are shown here. The open reading frame (ORF), gene name and a brief description of function are provided for each gene. Description of function was obtained from Saccharomyces Genome Database. [file FSN3-12-4927-s002.pdf]

Table S1. Z-scores reflecting the ratio of GFP fluorescence in treated and untreated cells of each protein that had a significant change in abundance due to Ech A treatment. The z-score was calculated every 30 minutes for 4 hours for 4,100 strains and only significant changes are shown here.

			Ech A treatment (minutes)								
**ORF**	**Protein**	**Description**	**0**	**30**	**60**	**90**	**120**	**150**	**180**	**210**	**240**
			**Increased protein abundance**								
YAL015C	NTG1	DNA N-glycosylase and apurinic/apyrimidinic (AP) lyase involved in base excision repair in response to oxidative stress	-	-	10.10	-	-	-	-	-	-
YBL013W	FMT1	Methionyl-tRNA formyltransferase in mitochondria	-	-	-	11.50	12.08	13.73	15.21	14.17	15.53
YBL080C	PET112	Subunit of the trimeric GatFAB AmidoTransferase(AdT) complex involved in the formation of Q-tRNAQ	-	-	-	-	-	11.35	-	-	10.80
YBR008C	FLR1	Plasma membrane multidrug transporter of the major facilitator superfamily	-	-	-	10.82	-	14.15	14.63	15.11	20.49
YBR093C	PHO5	Phosphatase that mediates extracellular nucleotide-derived phosphate hydrolysis	-	-	-	-	-	-	-	-	12.44
YBR138C	YBR138C	Cytoplasmic protein of unknown function	-	-	-	-	10.806	-	12.18	12.71	12.49
YBR151W	APD1	Protein of unknown function known to be required for normal tolerance of hydrogen peroxide	10.23	17.16	22.12	22.82	24.02	31.31	27.33	33.13	35.54
YBR207W	FTH1	Putative high affinity iron transporter involved in transport of intravacuolar stores of iron	-	-	-	-	11.49	-	10.47	12.60	10.14
YBR272C	HSM3	Proteasome-interacting protein involved in DNA mismatch repair and assembly of the 19S proteasome	-	-	12.36	-	-	-	-	-	-

YDL033C	SLM3	tRNA-specific 2-thiouridylase involved in 2-thiolation of the wobble base of mitochondrial tRNAs	-	-	-	-	-	-	-	10.67	11.63
YDL104C	QRI7	Highly conserved mitochondrial protein, required for t6A modification of mitochondrial tRNAs	-	10.58	-	-	-	-	- 10.29	-	-
YDL105W	NSE4	Nuclear protein that functions in the Smc5-Rhc18 complex	-	-	-	-	-	-	-	10.89	15.17
YDL108W	KIN28	Serine/threonine protein kinase that is a subunit of the transcription factor TFIIH involved in transcription initiation at RNA polymerase II promoters	-	-	-	-	-	-	-	-	11.11
YDR004W	RAD57	Recombinational repair of double-strand breaks in DNA during vegetative growth and meiosis	-	13.35	11.50	-	-	-	-	-	-
YDR011W	SNQ2	Plasma membrane multidrug transporter of the major facilitator superfamily involved in resistance to singlet oxygen species	-	16.74	21.15	29.99	31.45	33.80	43.21	50.21	51.25
YDR043C	NRG1	Transcriptional repressor that mediates glucose repression, filamentous growth and alkaline pH response	-	-	-	10.68	11.83	-	-	-	-
YDR105C	TMS1	Highly conserved vacuolar membrane protein of unknown function that is conserved in mammals	-	-	-	-	-	-	-	10.48	-
YDR132C	MRX16	Protein that interacts with the large mitoribosomal subunit and increases upon DNA replication stress	-	-	10.25	-	-	-	10.40	12.71	12.08
YDR191W	HST4	Member of the Sir2 family of NAD(+)-dependent protein deacetylases involved in silencing at telomeres, cell cycle progression, radiation resistance,	-	-	-	11.48	10.09	-	12.06	13.77	16.82

		genomic stability and short-chain fatty acid metabolism									
YDR270W	CCC2	Cu(+2)-transporting P-type ATPase required for export of copper from the cytosol	-	-	17.20	15.42	14.41	14.19	16.51	18.54	20.60
YDR460W	TFB3	Subunit of TFIIH involved in transcription initiation and nucleotide excision repair factor	-	-	-	-	-	-	10.23	-	10.64
YDR476C	YDR476C	Unknown function	-	-	14.62	15.53	16.60	20.21	23.94	23.06	26.96
YDR520C	URC2	Putative Zn(II)2Cys6 motif containing transcription factor; possibly involved in rDNA transcription and uracil catabolism	-	-	-	12.12	-	-	-	-	-
YEL029C	BUD16	Putative pyridoxal kinase required for genome stability and synthesis of the active form of vitamin B6	-	-	10.92	10.28	14.03	12.83	15.76	17.41	18.13
YER054C	GIP2	Putative regulatory subunit of the protein phosphatase Glc7p involved in glycogen metabolism	-	-	10.12	-	-	12.99	12.74	12.93	15.54
YER145C	FTR1	High affinity iron permease involved in the transport of iron across the plasma membrane	-	-	-	-	-	-	-	-	10.54
YFL027C	GYP8	GTPase-activating protein for yeast Rab family members; involved in the regulation of ER-to-Golgi transport	-	11.55	10.83	13.66	14.476	13.44	16.95	18.57	19.20
YFR046C	CNN1	Kinetochore protein of unknown function	-	11.11	10.14	-	11.18	-	-	-	11.83
YGL209W	MIG2	Zinc finger protein involved in glucose response	-	-	-	-	-	-	-	-	10.88
YGR017W	YGR017W	Putative protein of unknown function	-	-	-	-	-	-	11.14	-	-
YGR052W	FMP48	Putative mitochondrial protein of unknown function	-	10.19	10.16	10.57	-	11.46	10.94	11.29	11.51

YGR161C	RTS3	Putative member of the protein phosphatase type 2A complex	-	-	-	-	-	11.29	12.92	12.91	11.88
YGR243W	MPC3	Putative mitochondrial protein of unknown function	-	-	-	-	-	-	10.18	10.60	15.78
YGR257C	MTM1	Mitochondrial protein that activates mitochondrial superoxide dismutase involved in antioxidant activity	-	-	-	-	-	-	-	-	11.09
YGR281W	YOR1	Plasma membrane multidrug transporter of the major facilitator superfamily	-	-	-	12.77	14.97	17.52	17.59	22.64	27.68
YHL020C	OPI1	Transcriptional regulation of phospholipid biosynthesis	-	-	-	-	-	-	10.24	11.68	14.16
YHL040C	ARN1	Transporter of iron bound to ferrirubin, ferrirhodin, and related siderophores	-	-	11.91	13.56	15.626	16.39	15.02	19.45	21.48
YHR140W	YHR140W	Putative plasma membrane protein of unknown function	-	-	-	-	-	12.44	14.91	18.19	18.95
YIL104C	SHQ1	Chaperone protein involved in rRNA processing and the heat shock response	-	-	-	-	-	11.48	18.05	16.18	17.87
YIL160C	POT1	3-ketoacyl-CoA thiolase that cleaves 3-ketoacyl-CoA into acyl-CoA and acetyl-CoA during beta-oxidation of fatty acids	-	-	-	-	-	-	-	13.26	14.06
YJR043C	POL32	Third subunit of DNA polymerase delta involved in DNA damage and repair	-	11.25	-	-	-	-	-	-	-
YJR053W	BFA1	Component of the GTPase-activating Bfa1-Bub2 complex involved in mitosis	-	-	-	-	-	-	-	-	10.41
YJR136C	TTI2	Putative protein of unknown function; possibly involved in chromatin remodeling	-	-	-	-	10.65	11.85	10.68	-	11.68
YJR148W	BAT2	Cytosolic protein involved in branched chain amino acid catabolism	-	-	-	-	-	-	-	-	10.96
YKL103C	APE1	Vacuolar aminopeptidase protein involved in autophagy and the cytosol-to-vacuole targeting pathway	-	-	-	-	-	-	11.26	11.25	12.69

YKL108W	SLD2	Single-stranded DNA origin-binding and annealing protein required for the initiation of DNA replication	-	10.63	15.95	-	-	10.37	11.34	12.22	18.94
YKL162C	YKL162C	Putative mitochondrial protein of unknown function	10.87	10.21	-	10.41	14.710	13.81	10.46	-	11.06
YKR011C	YKR011C	Putative nuclear protein of unknown function	-	-	-	-	-	10.46	-	-	-
YKR052C	MRS4	Protein that transports iron across the inner mitochondrial membrane	-	-	12.07	14.26	13.63	14.50	12.31	12.21	18.15
YLR034C	SMF3	Putative divalent metal ion transporter involved in iron homeostasis	-	12.32	11.07	10.35	13.04	10.62	16.69	16.69	16.87
YLR085C	ARP6	Actin-related protein and component of the SWR1 complex that exchanges histone variant H2AZ (Htz1p) for chromatin-bound histone H2A	-	11.12	-	-	-	10.11	-	10.47	10.53
YLR105C	SEN2	Subunit of the tRNA splicing endonuclease	-	-	-	-	-	-	12.24	-	-
YLR107W	REX3	RNA exonuclease	-	11.04	-	-	-	-	-	-	-
YLR136C	TIS11	mRNA-binding protein expressed during iron starvation	15.46	25.52	17.95	18.81	14.14	17.16	22.24	25.14	28.71
YLR179C	YLR179C	Protein of unknown function	-	-	14.04	15.65	19.04	23.70	24.30	23.38	28.74
YLR265C	NEJ1	Protein involved in regulation of nonhomologous end joining	-	-	-	-	-	10.65	12.63	11.70	11.25
YLR346C	CIS1	Putative protein of unknown function localized to mitochondria; expression is regulated by transcription factors involved in pleiotropic drug resistance	-	14.47	20.66	29.04	33.58	39.58	40.43	44.76	49.16
YLR405W	DUS4	Dihydrouridine synthase	-	-	-	-	-	-	-	11.35	15.56
YML038C	YMD8	Putative nucleotide sugar transporter	-	-	10.79	12.38	19.29	19.81	17.49	18.63	20.96

YML060W	OGG1	Mitochondrial glycosylase/lyase that repairs DNA damage and oxidative stress	-	-	-	-	-	-	-	-	12.77
YML102W	CAC2	Highly conserved component of the chromatin assembly complex required for kinetochore function and assembly of histones onto replicated DNA	-	11.05	-	-	-	-	-	-	-
YMR021C	MAC1	Copper-sensing transcription factor that regulates genes required for high affinity copper transport	-	-	-	-	-	-	-	-	12.73
YMR067C	UBX4	UBX (ubiquitin regulatory X) domain-containing protein that interacts with Cdc48p	-	-	-	-	-	11.19	-	-	-
YMR102C	LAF1	Protein of unknown function	-	-	-	-	-	-	-	-	10.95
YMR171C	EAR1	Specificity factor required for Rsp5p-dependent ubiquitination and sorting of specific cargo proteins at the multivesicular body	-	-	-	-	-	11.22	-	-	-
YMR220W	ERG8	Phosphomevalonate kinase involved in the biosynthesis of isoprenoids and sterols from mevalonate	-	10.60	13.67	14.27	16.64	15.78	18.45	18.22	21.11
YMR253C	YMR253C	Putative cytoplasmic protein of unknown function	-	-	-	13.05	-	13.71	12.97	10.90	13.85
YMR287C	DSS1	3'-5' exoribonuclease and component of the mitochondrial degradosome	-	-	-	-	-	-	10.87	-	10.18
YNL164C	IBD2	Component of the BUB2-dependent spindle checkpoint pathway	-	10.31	-	-	-	-	-	-	-
YNL217W	PPN2	Putative vacuolar protein of unknown function	-	-	-	-	-	-	-	10.00	-
YNL239W	LAP3	Cysteine aminopeptidase with homocysteine-thiolactonase activity; protection against homocysteine toxicity	-	-	-	-	-	10.95	10.24	10.84	12.17

YNL254C	RTC4	Protein of unknown function localized to the cytoplasm and nucleus	-	-	-	-	-	-	-	-	11.52
YNL305C	BXI1	Protein localized to the ER and vacuole that functions in apoptosis	-	-	-	-	-	-	10.13	-	-
YOL049W	GSH2	Glutathione synthetase induced by oxidative stress and heat shock	-	-	-	-	-	10.58	12.59	11.32	13.02
YOL082W	ATG19	Receptor protein required for the cytoplasm-to-vacuole targeting (Cvt) pathway	-	10.25	-	-	-	-	-	-	-
YOR059C	LPL1	Lipid particle protein of unknown function	-	-	-	12.84	13.82	17.52	15.09	18.07	14.18
YOR065W	CYT1	Cytochrome c1 that is a component of the mitochondrial respiratory chain; regulated by heme and glucose	-	-	-	-	-	10.88	13.00	15.57	15.08
YOR316C	COT1	Protein that transports zinc into the vacuole	-	-	-	-	-	10.58	11.69	13.81	14.43
YOR324C	FRT1	Tail-anchored ER membrane protein that is a substrate of the phosphatase calcineurin	-	-	-	10.41	12.38	14.13	11.08	13.66	-
YPL026C	SKS1	Putative glucose-responsive serine/threonine protein kinase	-	-	-	-	-	-	10.67	-	-
YPL060W	MFM1	Protein that regulates magnesium levels in the mitochondria via transport	11.75	-	-	-	-	-	-	10.78	11.85
YPL135W	ISU1	Conserved protein of the mitochondrial matrix involved in the assembly of iron-sulfur clusters	-	-	-	-	-	-	-	-	10.15
YPL139C	UME1	Negative regulator of meiosis viabinding with histone deacetylase Rpd3	-	-	10.70	-	-	-	-	-	12.28
YPL152W	RRD2	Activator of phosphatase activity and regulator of G1 phase progression, osmoresponse and microtubule dynamics	-	10.76	-	-	-	-	-	-	-

YPL170W	DAP1	Heme-binding protein that regulates sterol synthesis	-	11.79	13.75	19.59	23.30	30.03	33.16	39.98	40.18
YPL209C	IPL1	Component of the conserved chromosomal passenger complex involved in regulating kinetochore-microtubule attachments	10.44	-	-	-	-	13.30	18.07	13.61	15.01
		**Decreased protein abundance**									
YCR087C-A	YCR087C-A	Putative protein of unknown function localized to the nucleolus	-	-10.40	-14.91	-11.50	-15.90	-18.85	-15.66	-17.99	-16.52
YDL104C	QRI7	Highly conserved mitochondrial protein required for modification of mitochondrial tRNAs	-	10.58	-	-	-	-	-10.29	-	-
YGR138C	TPO2	Plasma membrane protein specific for transport of the polyamine spermine	-	-	-	-	-	-	-	-10.79	-13.34
YGR280C	PXR1	Essential protein involved in rRNA and snoRNA maturation	-	-	-	-	-	-	-11.96	-	-
YNL065W	AQR1	Plasma membrane multidrug transporter of the major facilitator superfamily	-	-	-	-	-	-11.43	-13.35	-14.06	-15.00
YPL274W	SAM3	High-affinity S-adenosylmethionine permease	-	-	-	-11.00	-13.069	-16.45	-19.49	-23.48	-25.73

Table S2. Annotation of mitochondrial proteins that were significantly altered in abundance due to Ech A treatment. The open reading frame (ORF), protein name and a brief description of function are provided for each protein. Description of function was obtained from Saccharomyces Genome Database.

ORF	Gene	Description
YNL305C	BXI1	Protein localized to ER and vacuole that translocates to mitochondria under apoptosis-inducing conditions
YLR346C	CIS1	Putative protein of unknown function localized to mitochondria; expression is regulated by transcription factors involved in pleiotropic drug resistance
YOR065W	CYT1	Cytochrome c1 that is a component of the mitochondrial respiratory chain; regulated by heme and glucose
YPL170W	DAP1	Heme-binding protein that regulates sterol synthesis
YMR287C	DSS1	3'-5' exoribonuclease and component of the mitochondrial degradosome
YGR052W	FMP48	Putative mitochondrial protein of unknown function
YBL013W	FMT1	Methionyl-tRNA formyltransferase in mitochondria
YPL135W	ISU1	Conserved protein of the mitochondrial matrix involved in the assembly of iron-sulfur clusters
YPL060W	MFM1	Protein that regulates magnesium levels in the mitochondria via transport
YGR243W	MPC3	Putative mitochondrial protein of unknown function
YKR052C	MRS4	Protein that transports iron across the inner mitochondrial membrane
YGR257C	MTM1	Mitochondrial protein that activates mitochondrial superoxide dismutase involved in antioxidant activity
YML060W	OGG1	Mitochondrial glycosylase/lyase that repairs DNA damage and oxidative stress
YDL104C	QRI7	Highly conserved mitochondrial protein, required for t6A modification of mitochondrial tRNAs
YDL033C	SLM3	tRNA-specific 2-thiouridylase involved in 2-thiolation of the wobble base of mitochondrial tRNAs

Table S3. Annotation of proteins involved in drug response that were significantly altered in abundance due to Ech A treatment. gene deletion strains sensitive to Ech A. The open reading frame (ORF), protein name and a brief description of function are provided for each protein. Description of function was obtained from Saccharomyces Genome Database.

ORF	Gene	Description
YDR011W	SNQ2	Plasma membrane multidrug transporter of the major facilitator superfamily involved in resistance to singlet oxygen species
YGR281W	YOR1	Plasma membrane multidrug transporter of the major facilitator superfamily
YBR008C	FLR1	Plasma membrane multidrug transporter of the major facilitator superfamily
YLR179C	YLR179C	Protein of unknown function
YLR346C	CIS1	Putative protein of unknown function localized to mitochondria; expression is regulated by transcription factors involved in pleiotropic drug resistance

Table S4. Annotation of proteins involved in metal ion metabolism that were significantly altered in abundance due to Ech A treatment. gene deletion strains sensitive to Ech A. The open reading frame (ORF), protein name and a brief description of function are provided for each protein. Description of function was obtained from Saccharomyces Genome Database.

ORF	Gene	Description
YPL170W	DAP1	Heme-binding protein that regulates sterol synthesis
YOR065W	CYT1	Cytochrome c1 that is a component of the mitochondrial respiratory chain; regulated by heme and glucose
YHL040C	ARN1	Transporter of iron bound to ferrirubin, ferrirhodin, and related siderophores
YLR136C	TIS11	mRNA-binding protein expressed during iron starvation
YKR052C	MRS4	Protein that transports iron across the inner mitochondrial membrane
YLR034C	SMF3	Putative divalent metal ion transporter involved in iron homeostasis
YDR270W	CCC2	Cu(+2)-transporting P-type ATPase required for export of copper from the cytosol
YOR316C	COT1	Protein that transports zinc into the vacuole

Table S5. Annotation of gene deletion strains sensitive to Ech A. The open reading frame (ORF), gene name and a brief description of function are provided for each gene. Description of function was obtained from Saccharomyces Genome Database.

ORF	Gene	Description
YBR281C	DUG2	Probable di- and tri-peptidase involved in the degradation of glutathione (GSH)
YCR081W	SRB8	Subunit of the RNA polymerase II mediator complex required for transcriptional regulation
YDL142C	CRD1	Cardiolipin Major synthase gene required for biosynthesis of cardiolipin in mitochondrial inner membrane
YDR207C	UME6	Key transcriptional regulator of early meiotic genes responsive to nutritional cues with initiation and progression of meiosis
YDR315C	IPK1	Nuclear-localized inositol 1,3,4,5,6-pentakisphosphate 2-kinase required for synthesis of 1,2,3,4,5,6-hexakisphosphate (phytate) that is integral to cell function
YFL014W	HSP12	Plasma membrane protein involved in maintaining membrane organization in stress conditions such as heat shock, oxidative stress, fatty acid stress and glucose depletion
YGL168W	HUR1	Protein of unknown function
YHR008C	SOD2	Mitochondrial manganese superoxide dismutase with antioxidant activity
YHR021C	RPS27B	Component of the small (40S) ribosomal subunit
YHR030C	SLT2	Serine/threonine MAP kinase that regulates maintenance of cell wall integrity in diverse processes such as the cell cycle, heat shock response, mitophagy and pexophagy
YKL023W	SKA1	Putative protein of unknown function possibly involved in mRNA degradation
YKL037W	AIM26	Putative protein of unknown function possibly involved in mitochondrial genome stability
YKL041W	VPS24	Component of the endosomal sorting complex involved in the sorting of transmembrane proteins into the multivesicular body (MVB) pathway
YKR024C	DBP7	Putative ATP-dependent RNA helicase of the DEAD-box family involved in ribosomal biogenesis
YLR124W	YLR124W	Dubious open reading frame unlikely to encode a protein

YLR425W	TUS1	Guanine nucleotide exchange factor (GEF) that modulates Rho1 activity as part of the cell integrity signaling pathway
YMR031W-A	YMR031W-A	Dubious open reading frame unlikely to encode a protein
YMR202W	ERG2	C-8 sterol isomerase required for an intermediate step in ergosterol biosynthesis
YOR035C	SHE4	Myosin-binding protein involved in endocytosis, polarization of the actin cytoskeleton, and asymmetric mRNA localization
YOR162C	YRR1	Zinc-finger transcription factor that activates genes involved in multidrug resistance
